# Supplementary material for: Improving the phenotype predictions of a yeast genome‐scale metabolic model by incorporating enzymatic constraints
Source: Mol Syst Biol. 2017 Aug 4;13(8):935. doi: 10.15252/msb.20167411 (PMC5572397; doi:10.15252/msb.20167411)
Supplement: Supplementary file 1 — Appendix [file MSB-13-935-s001.pdf]

Appendix

Improving the phenotype predictions of a yeast genome-scale metabolic model by incorporating enzymatic constraints

Benjamín J. Sánchez[1,2(#)], Cheng Zhang[3,4(#)], Avlant Nilsson[1], Petri-Jaan Lahtvee[1,2], Eduard J. Kerkhoven[1,2] and Jens Nielsen[1,2,5(*)]

[1] Department of Biology and Biological Engineering, Chalmers University of Technology, SE41296 Gothenburg, Sweden

[2] Novo Nordisk Foundation Center for Biosustainability, Chalmers University of Technology, SE41296 Gothenburg, Sweden

[3] Science for Life Laboratory, KTH - Royal Institute of Technology, SE17121 Stockholm, Sweden

[4] State Key Laboratory of Bioreactor Engineering, East China University of Science and Technology, Shanghai, China

[5] Novo Nordisk Foundation Center for Biosustainability, Technical University of Denmark, DK2970 Hørsholm, Denmark

(#) Shared first authorship.

(*) Corresponding author.

Correspondence: nielsenj@chalmers.se

Table of contents

List of tables *3*

List of figures *4*

1. Framework development *5*

1.1. Flux balance analysis: Quick recap 5

1.2. Constraints connecting enzymes with reactions 5

1.3. Including enzymes in reactions 6

1.4. Example with a toy model 10

1.5. Going genome-scale 14

2. Description of the method *16*

2.1. Retrieving enzyme data 16

2.1.1. Retrieving k_{cat} values: querying BRENDA 16

2.1.2. Retrieving other enzyme data: querying SWISS-PROT and KEGG 17

2.2. Genome-scale model pre-processing 18

2.2.1. Model corrections 18

2.2.2. Standardization of model 20

2.3. Matching k_{cat} data to the model 21

2.3.1. Matching reactions to enzymes 21

2.3.2. Automatic k_{cat} matching criteria 22

2.3.3. Further manual curation of enzyme data 23

2.4. Adding enzymes to the model 26

2.5. Constraining enzyme levels in the model 28

2.5.1. In case of no protein data: the 'pool' assumption 30

3. Additional methodology caveats *32*

3.1. Visualization caveats 32

3.1.1. Classification of enzymes by metabolic group 32

3.1.2. Connectivity of model 33

3.2. Simulation caveats 35

3.2.1. Chemostat growth 35

3.2.2. Batch growth 36

3.2.3. Flux variability analysis 36

3.2.4. Random sampling 37

3.2.5. Anaerobic growth in glucose-limited chemostats 38

3.2.6. Proteomic integration 39

3.2.7. Flux control coefficient analysis 40

4. Supplementary results *41*

4.1. Description of the model 41

4.2. Enzyme features 43

4.3. Connectivity of model 47

4.4. Chemostats with varying dilution rate 47

4.5. Growth under temperature stress 51

4.6. Proteomic integration 52

4.7. *NDI1* knockout study 55

4.8. Flux control coefficients for farnesene production 56

5. Supplementary references *57*

List of tables

Table S1: Original and corrected coefficients in the carbohydrate composition of the biomass pseudo-reaction of Yeast 7 19

Table S2: KEGG pathways classified by three metabolic groups: Carbohydrate and energy primary metabolism (CE), aminoacid, fatty acid and nucleotide primary metabolism (AFN), and intermediate and secondary metabolism (IS). 32

Table S3: Number of k_{cat} values obtained by using different criteria with the GECKO toolbox. 41

Table S4: p-values across metabolic groups, for both k_{cat} values and molecular weights. CE = carbohydrate and energy primary metabolism. AFN = amino acid, fatty acid and nucleotide primary metabolism. IS = intermediate and secondary metabolism. 44

Table S5: Comparison of predicted flux ratios by Yeast7 and ecYeast7 to experimental values attained by ^{13}C-MFA from a previous study[50]. The predicted values were calculated by adding up the fluxes corresponding to the reaction sets shown in the 4th column. If a reaction had a reversible counterpart the corresponding flux was subtracted, except for the OAA transport (as both directions are present in the study). The metabolite sets G3P/3PG, Ru5P/Ro5P/X5P and OAA/MAL (the latter both in cytoplasm and mitochondria) were considered as one metabolite each, in order to adapt to the model proposed in the study with the experimental data[50]. 53

List of figures

Figure S1: Toy model used for the example. 10

Figure S2: Histograms of the model. (A) Number of subunits per complex. (B) Number of isozymes per reaction with isozymes. (C) Number of reactions per promiscuous enzyme. (D) Number of reactions per promiscuous enzyme (zoom). 42

Figure S3: Overview of enzymatic data in model. (A) Histogram of enzymes' molecular weights. (B) Semi-logarithmic plot between enzymes' molecular weights and enzymes' k_{cat} values, showing that there is no strong linear correlation between both variables (red line, $R^2 = 0.010$). (C) Logarithmic histogram of enzymes' k_{cat} values. 43

Figure S4: Cumulative distributions of k_{cat} values of the model's enzymes. Note that the scale is logarithmic. (A) All enzymes in model. (B) Proteins part of complexes (blue) Vs standalone enzymes (grey). (C) Enzymes catalyzing reactions with isozymes (red) Vs enzymes catalyzing reactions with no isozymes (grey). (D) Promiscuous enzymes (green) Vs non-promiscuous enzymes (grey). 45

Figure S5: Cumulative distributions of molecular weights of the model's enzymes. (A) All enzymes in model. (B) Proteins part of complexes (blue) Vs standalone enzymes (grey). (C) Enzymes catalyzing reactions with isozymes (red) Vs enzymes catalyzing reactions with no isozymes (grey). (D) Promiscuous enzymes (green) Vs non-promiscuous enzymes (grey). 46

Figure S6: Node degree histograms of original metabolic model (blue) and enzyme-constrained model (green), (A) with and (B) without currency metabolites. 47

Figure S7: Model predictions (lines) and experimental values (points) for exchange fluxes from glucose-limited chemostats: glucose (green circles) and oxygen (blue squares) consumption, and CO_2 (purple diamonds), ethanol (red triangles), acetate (orange inverted triangles) and glycerol (yellow right-pointing triangles) production. (A) Yeast7; aerobic conditions. (B) ecYeast7; aerobic conditions (the light blue area denotes the region of both glucose and protein limitation). (C) Yeast7; anaerobic conditions. (D) ecYeast7; anaerobic conditions. 48

Figure S8: Performance of enzyme-constrained models with random k_{cat} values (blue) and molecular weights (green). In yellow the original selection for ecYeast7 is shown. (A) Overall fitting error to the experimental data (aerobic chemostats at increasing dilution rate[41]). (B) Dilution rate at which the protein content becomes limited. (C) Oxygen consumption rate at a dilution rate of 0.4 h^{-1}. (D) Ethanol production rate at a dilution rate of 0.4 h^{-1}. 49

Figure S9: Cumulative distribution of fluxes with non-zero variability for Yeast7 (red) and ecYeast7 (blue), under (A) low specific growth rate = 0.025 h^{-1} and (B) high specific growth rate = 0.4 h^{-1}. 50

Figure S10: Both Yeast7 and ecYeast7 miss predict temperature stress experimental data when NGAM is unchanged. 51

Figure S11: Flux predictions of the original metabolic model (Yeast7) and the enzyme constrained model (ecYeast7) compared to experimental data of yeast grown at 0.1 h^{-1} aerobically limited on glucose. 52

Figure S12: Performance of both Yeast7 (red) and ecYeast7 (blue) predicting intracellular flux ratios from central carbon metabolism. Experimental values obtained from a published ^{13}C-MFA study[50]. 54

Figure S13: Histogram of reactions with (A) increased and (B) decreased flux variability. 54

Figure S14: Model predictions (lines) and experimental values (points) for exchange fluxes from glucose-limited chemostats: glucose (green circles) and oxygen (blue squares) consumption, and CO_2 (purple diamonds), ethanol (red triangles) and glycerol (yellow right-pointing triangles) production. The light blue area denotes the region of both glucose and protein limitation. (A) Wild-type strain. (B) NDI1 knockout 55

Figure S15: Flux control coefficients of the top 10 most influential enzymes in the model. 56

1. Framework development

1.1. Flux balance analysis: Quick recap

Flux balance analysis (FBA) has been reviewed elsewhere[1] and is based on the pseudo-steady state assumption, i.e. that under short timescales there is no accumulation of intracellular metabolites. Therefore a mass balance of all metabolites yield

$$\mathrm{S} \cdot \mathrm{v} = 0 \qquad [\mathrm{Eq. S1}]$$

Where S is the stoichiometric matrix, which contains the stoichiometric coefficients for all reactions (as columns) and metabolites (as rows), and v is the vector of all metabolic fluxes (mmol/gDWh). Because the matrix S usually has more columns than rows, equation S1 has multiple solutions, so additional constraints are needed to solve the system. The fluxes can be constrained if reversibility/irreversibility of reactions are known, and if some uptake/production rates are measured. These constraints are represented as:

$$\mathrm{LB} \leq \mathrm{v} \leq \mathrm{UB} \qquad [\mathrm{Eq. S2}]$$

Where LB and UB are the lower and upper bound vectors containing the constraints for each flux. Equations S1 and S2 define a subspace of feasible solutions, and if an objective function is assumed, an optimal solution can be found.

1.2. Constraints connecting enzymes with reactions

Our aim in this study is to model with proper constraints the relationship between enzymes and reactions in the cell, to further constrain flux vector v. For this, we will start with the simplest scenario: an enzyme $\mathrm{E_i}$ which catalyzes only one reaction $\mathrm{R_j}$ (i.e. no promiscuity), reaction which is in turn only catalyzed by enzyme $\mathrm{E_i}$ (i.e. no isozymes). For this pair enzyme/reaction (assuming that the reaction is irreversible), it holds true that:

$$\mathrm{v_j} \leq \mathrm{k_{cat}^{ij}} \cdot [\mathrm{E_i}] \qquad [\mathrm{Eq. S3}]$$

Here $\mathrm{v_j}$ is the flux through reaction $\mathrm{R_j}$ (mmol/gDWh), $\mathrm{k_{cat}^{ij}}$ is the turnover number (i.e. maximum specific rate; $\mathrm{h^{-1}}$) and $[\mathrm{E_i}]$ is the concentration of the enzyme (mmol/gDW). Note that the right side

of the equation is the maximum flux (v_{max}). Also note that k_{cat}^{ij} will depend on both the enzyme and the reaction, because the binding enzyme-substrate could be different.

Equation S3 is the simplest scenario, but more complicated relationships are quite common in metabolism. For isozymes, i.e. several enzymes that can catalyze the same reaction, the relationship would be:

$$v_j \leq \sum_i k_{cat}^{ij} \cdot [E_i] \qquad [Eq. S4]$$

For a promiscuous enzyme, i.e. an enzyme that can catalyze several different reactions:

$$\sum_j \frac{v_j}{k_{cat}^{ij}} \leq [E_i] \qquad [Eq. S5]$$

Finally, for a complex, i.e. a set of subunits that together work as a whole enzyme:

$$v_j \leq k_{cat}^{ij} \cdot \underset{k}{Min}\left(\frac{[U_{ik}]}{s_{ik}}\right) \qquad [Eq. S6]$$

Where s_{ik} is the stoichiometry and $[U_{ik}]$ the concentration (mmol/gDW) of subunit U_{ik}, part of enzyme E_i. Even more complicated relationships emerge if some of this rules are combined; for instance, a promiscuous enzyme could catalyze one reaction that is also catalyzed by an alternative isozyme. Therefore, an approach in which this relationships can be decomposed is needed.

1.3. Including enzymes in reactions

In our approach, we include enzymes as part of the reactions in the model. For a generic reaction such as the following:

$$R_j: \quad A + B \xrightarrow{E_i} C + D \qquad [Eq. S7]$$

Which is catalyzed by enzyme E_i, we transform it to:

$$R_j: \quad n_{ij}E_i + A + B \rightarrow C + D \qquad [Eq. S8]$$

With a reaction/enzyme specific stoichiometric coefficient n_{ij} to be determined. Note that even though enzymes are not consumed in reactions (because they are catalysts), for a short period of time they are being occupied, so one should interpret the enzyme "consumption" in equation S8 as the usage of an amount of enzyme at a specific time to catalyze the flux going through reaction R_j. Therefore, the enzyme in the reaction is treated as a pseudo-metabolite and does not affect the mass balance of the reaction.

Because mass balances of enzymes should also be respected, by including E_i in the reaction (that will be used if the reaction carries flux), we should include an overall enzyme usage pseudo-reaction EU_i that supplies the cell with the corresponding amount of enzyme:

$$\mathrm{EU_i}: \quad \rightarrow \mathrm{E_i} \qquad [\mathrm{Eq.S9}]$$

If we call e_i the "flux" carried by this pseudo-reaction, then it can be constrained with the enzyme's concentration (mmol/gDW):

$$0 \le \mathrm{e_i} \le [\mathrm{E_i}] \qquad [\mathrm{Eq.S10}]$$

Note that the units here do not correspond with the typical units of flux (mmol/gDWh), and this is because EU_i is not a real reaction but just a mathematical construct to represent the enzyme's usage in the model. Additionally, because we are working under a steady state assumption, we are just observing a specific time point of metabolism, therefore we do not take into account production and degradation of the enzyme, but only the enzyme usage for catalyzing the corresponding reactions. We can then define a mass balance for enzyme E_i (considering equations S8 and S9):

$$-\mathrm{n_{ij}} \cdot \mathrm{v_j} + \mathrm{e_i} = 0 \qquad [\mathrm{Eq.S11}]$$

Combining equations S10 and S11 and rearranging we get:

$$\mathrm{v_j} \le \frac{1}{\mathrm{n_{ij}}} \cdot [\mathrm{E_i}] \qquad [\mathrm{Eq.S12}]$$

Comparing this equation to equation S3 we conclude that the stoichiometric coefficient for the enzyme E_i in reaction R_j should be:

$$\mathrm{n_{ij}} = \frac{1}{\mathrm{k_{cat}^{ij}}} \qquad [\mathrm{Eq.S13}]$$

Therefore, the modifications that should be performed to account for an enzymatic constraint in FBA should be (a) include the enzyme as a metabolite in the corresponding reaction with the inverted k_{cat} as the stoichiometric coefficient, (b) include an enzyme usage pseudo-reaction for the enzyme, and (c) define an upper bound for the enzyme usage equal to the measured concentration of that enzyme.

Some additional considerations should be taken in specific cases:

- Reversible reactions: The transformation shown in equation S8 only works if all reactions are defined as irreversible. Therefore, in the case that the reaction shown in equation S7 was a reversible reaction, 2 reactions should be instead defined, one in the forward direction ($R_{j/f}$) and one in the backward direction ($R_{j/b}$), both with the same enzyme as substrate, but possibly with different k_{cat} values, depending on the substrate affinity:

$$R_{j/f}: \quad \frac{1}{k_{cat}^{ij/f}} E_i + A + B \rightarrow C + D \qquad [\mathrm{Eq.S14}]$$

$$R_{j/b}: \quad \frac{1}{k_{cat}^{ij/b}} E_i + C + D \rightarrow A + B \qquad [\mathrm{Eq.S15}]$$

- Isozymes: In the case of isozymes, because all isozymes would be equally capable of catalyzing the corresponding reaction, 1 reaction for each enzyme should be defined. For instance, if the reaction shown in equation S7 had 2 isozymes (E_1 and E_2), then the new reactions would be:

$$R_{j/1}: \quad \frac{1}{k_{cat}^{1j}} E_1 + A + B \rightarrow C + D \qquad [\mathrm{Eq.S16}]$$

$$R_{j/2}: \quad \frac{1}{k_{cat}^{2j}} E_2 + A + B \rightarrow C + D \qquad [\mathrm{Eq.S17}]$$

Note that the k_{cat} values can be different (because the enzyme is different). Also, in order to keep the same original upper bound in the reaction, we must introduce an "arm reaction"[2]: we create a pseudo-metabolite M_{int} that acts as an intermediate between the substrates and the products. The final formulation for isozymes then would consist of 3 reactions: 1

reaction from the substrates to the intermediate metabolite ($R_{j/arm}$), and 2 going from the intermediate product to the products, each using a different enzyme ($R_{j/1}$ and $R_{j/2}$). The original upper bound can that way still be respected by imposing it on reaction $R_{j/arm}$.

$$R_{j/arm}: \quad A + B \rightarrow M_{int} \qquad [Eq. S18]$$

$$R_{j/1}: \quad \frac{1}{k_{cat}^{1j}} E_1 + M_{int} \rightarrow C + D \qquad [Eq. S19]$$

$$R_{j/2}: \quad \frac{1}{k_{cat}^{2j}} E_2 + M_{int} \rightarrow C + D \qquad [Eq. S20]$$

- Promiscuous enzymes: For promiscuous enzymes there is no additional action needed: If a given enzyme takes part of 2 different reactions, then the same enzyme should be a substrate in both reactions. Note that only one enzyme usage pseudo-reaction will be defined, so both reactions will "share" the amount of enzyme available. Also note that the k_{cat} values can be different (because the substrate is different).
- Complexes: Finally, in the case of complexes all proteins are part of the same group that has the catalytic activity. The reaction then uses all proteins and shares the same k_{cat} value, but multiplied by the corresponding stoichiometry. As an example, if the reaction shown in equation S7 was catalyzed by a complex of 2 subunits (E_1 and E_2), then the reaction would be:

$$R_j: \quad \frac{s_1}{k_{cat}^{ij}} E_1 + \frac{s_2}{k_{cat}^{ij}} E_2 + A + B \rightarrow C + D \qquad [Eq. S21]$$

Where s_1 and s_2 are the corresponding stoichiometry of the subunits.

1.4. Example with a toy model

In the following we present an example on how our approach of additional constraints will work for a small network. Figure S1 shows the chosen model; it includes 3 exchange reactions (2 consumptions and 1 production) and 3 metabolic reactions. There is 1 reversible reaction, 1 complex (E_3 and $2E_4$), 1 reaction catalyzed by 2 possible enzymes (E_1 or E_2) and 1 promiscuous enzyme (E_1).

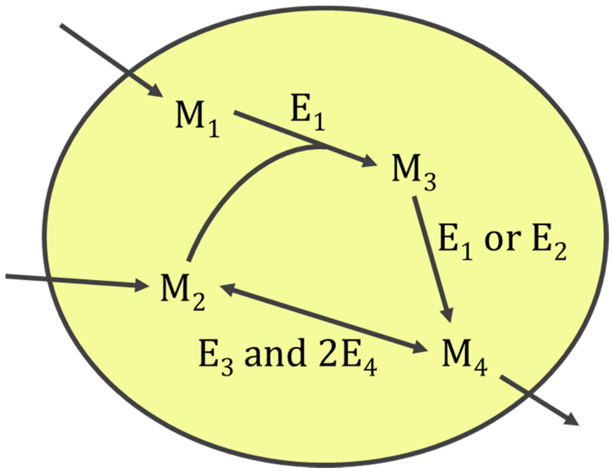

Figure S1: Toy model used for the example.

The 6 reactions (and the corresponding bounds for each flux) are the following:

$$R_1: \ \rightarrow M_1$$

$$R_2: \ \rightarrow M_2$$

$$R_3: \ M_1 + M_2 \xrightarrow{E_1} M_3$$

$$R_4: \ M_3 \xrightarrow{E_1 \text{ or } E_2} M_4$$

$$R_5: \ M_2 \xleftrightarrow{E_3 \text{ and } 2E_4} M_4$$

$$R_6: \ M_4 \rightarrow$$

$$0 \leq v_i \leq 1000 \quad ; \quad i = \{1, 2, 3, 4, 6\}$$

$$-1000 \leq v_5 \leq 1000$$

As stated previously, all reactions should be irreversible, therefore we first have to replace reaction R_5 by two irreversible reactions $R_{5/f}$ and $R_{5/b}$. Note that now all fluxes should be positive:

$$R_1: \rightarrow M_1$$

$$R_2: \rightarrow M_2$$

$$R_3:\ M_1 + M_2 \xrightarrow{E_1} M_3$$

$$R_4:\ M_3 \xrightarrow{E_1 \text{ or } E_2} M_4$$

$$R_{5/f}:\ M_2 \xrightarrow{E_3 \text{ and } 2E_4} M_4$$

$$R_{5/b}:\ M_4 \xrightarrow{E_3 \text{ and } 2E_4} M_2$$

$$R_6:\ M_4 \rightarrow$$

$$0 \leq v_i \leq 1000 \quad ; \quad i = \{1, 2, 3, 4, 5/f, 5/b, 6\}$$

Additionally, reaction R_4 has isozymes, therefore should be replaced by an arm reaction ($R_{4/arm}$) and 2 parallel reactions ($R_{4/1}$ and $R_{4/2}$):

$$R_1: \rightarrow M_1$$

$$R_2: \rightarrow M_2$$

$$R_3:\ M_1 + M_2 \xrightarrow{E_1} M_3$$

$$R_{4/arm}:\ M_3 \rightarrow PM_1$$

$$R_{4/1}:\ PM_1 \xrightarrow{E_1} M_4$$

$$R_{4/2}:\ PM_1 \xrightarrow{E_2} M_4$$

$$R_{5/f}:\ M_2 \xrightarrow{E_3 \text{ and } 2E_4} M_4$$

$$R_{5/b}:\ M_4 \xrightarrow{E_3 \text{ and } 2E_4} M_2$$

$$R_6:\ M_4 \rightarrow$$

$$0 \leq v_i \leq 1000 \quad ; \quad i = \{1, 2, 3, 4/arm, 4/1, 4/2, 5/f, 5/b, 6\}$$

Now we can perform the conversion of enzymes to metabolites, by adding them as substrates to the reactions and including the corresponding enzyme usage pseudo-reactions:

$$R_1:\ \rightarrow M_1$$

$$R_2:\ \rightarrow M_2$$

$$R_3:\ M_1 + M_2 + \frac{1}{k_{cat}^{13}} E_1 \rightarrow M_3$$

$$R_{4/arm}:\ M_3 \rightarrow PM_1$$

$$R_{4/1}:\ PM_1 + \frac{1}{k_{cat}^{14}} E_1 \rightarrow M_4$$

$$R_{4/2}:\ PM_1 + \frac{1}{k_{cat}^{24}} E_2 \rightarrow M_4$$

$$R_{5/f}:\ M_2 + \frac{1}{k_{cat}^{5/f}} E_3 + \frac{2}{k_{cat}^{5/f}} E_4 \rightarrow M_4$$

$$R_{5/b}:\ M_4 + \frac{1}{k_{cat}^{5/b}} E_3 + \frac{2}{k_{cat}^{5/b}} E_4 \rightarrow M_2$$

$$R_6:\ M_4 \rightarrow$$

$$ER_i:\ \rightarrow E_1 \quad ; \quad i = \{1, 2, 3, 4\}$$

$$0 \leq v_i \leq 1000 \quad ; \quad i = \{1, 2, 3, 4/arm, 4/1, 4/2, 5/f, 5/b, 6\}$$

$$0 \leq e_i \leq [E_i] \quad ; \quad i = \{1, 2, 3, 4\}$$

We now have 13 reactions (3 metabolite exchange reactions, 6 metabolic reactions and 4 enzyme usage pseudo-reactions) and 9 metabolites (4 real metabolites, 1 pseudo-metabolite and 4 enzymes). Note that k_{cat}^{14} and k_{cat}^{24} are different, because they are different enzymes, but there is just

one $k_{cat}^{5/f}$, because both subunits in $R_{5/f}$ act together as the same enzyme. The same can be said about $k_{cat}^{5/b}$.

We can now proceed to formulate the mass balances for all 9 metabolites:

$$M_1:\ v_1 - v_3 = 0$$

$$M_2:\ v_2 - v_3 - v_{5/f} + v_{5/b} = 0$$

$$M_3:\ v_3 - v_{4/arm} = 0$$

$$M_4:\ v_{4/1} + v_{4/2} + v_{5/f} - v_{5/b} - v_6 = 0$$

$$PM_1:\ v_{4/arm} - v_{4/1} - v_{4/2} = 0$$

$$E_1:\ -\frac{1}{k_{cat}^{13}} v_3 - \frac{1}{k_{cat}^{14}} v_{4/1} + e_1 = 0$$

$$E_2:\ -\frac{1}{k_{cat}^{24}} v_{4/2} + e_2 = 0$$

$$E_3:\ -\frac{1}{k_{cat}^{5/f}} v_{5/f} - \frac{1}{k_{cat}^{5/b}} v_{5/b} + e_3 = 0$$

$$E_4:\ -\frac{2}{k_{cat}^{5/f}} v_{5/f} - \frac{2}{k_{cat}^{5/b}} v_{5/b} + e_4 = 0$$

Note that if one combines the enzymes' mass balances together with the upper bounds of the enzymes' usage, one obtains the correct constraints relating fluxes and enzymes:

$$E_1:\ \frac{1}{k_{cat}^{13}} v_3 + \frac{1}{k_{cat}^{14}} v_{4/1} \leq [E_1]$$

$$E_2:\ \frac{1}{k_{cat}^{24}} v_{4/2} \leq [E_2]$$

$$E_3:\ \frac{1}{k_{cat}^{5/f}} v_{5/f} + \frac{1}{k_{cat}^{5/b}} v_{5/b} \leq [E_3]$$

$$E_4: \frac{2}{k_{cat}^{5/f}} v_{5/f} + \frac{2}{k_{cat}^{5/b}} v_{5/b} \leq [E_4]$$

Which was our original objective. Also, note that all mass balances can be formulated using matrix notation:

$$\begin{bmatrix} +1 & 0 & -1 & 0 & 0 & 0 & 0 & 0 & 0 & 0 & 0 & 0 & 0 \\ 0 & +1 & -1 & 0 & 0 & 0 & -1 & +1 & 0 & 0 & 0 & 0 & 0 \\ 0 & 0 & +1 & -1 & 0 & 0 & 0 & 0 & 0 & 0 & 0 & 0 & 0 \\ 0 & 0 & 0 & 0 & +1 & +1 & +1 & -1 & -1 & 0 & 0 & 0 & 0 \\ 0 & 0 & 0 & +1 & -1 & -1 & 0 & 0 & 0 & 0 & 0 & 0 & 0 \\ 0 & 0 & -\frac{1}{k_{cat}^{13}} & 0 & -\frac{1}{k_{cat}^{14}} & 0 & 0 & 0 & 0 & +1 & 0 & 0 & 0 \\ 0 & 0 & 0 & 0 & 0 & -\frac{1}{k_{cat}^{24}} & 0 & 0 & 0 & 0 & +1 & 0 & 0 \\ 0 & 0 & 0 & 0 & 0 & 0 & -\frac{1}{k_{cat}^{5/f}} & -\frac{1}{k_{cat}^{5/b}} & 0 & 0 & 0 & +1 & 0 \\ 0 & 0 & 0 & 0 & 0 & 0 & -\frac{2}{k_{cat}^{5/f}} & -\frac{2}{k_{cat}^{5/b}} & 0 & 0 & 0 & 0 & +1 \end{bmatrix} \bullet \begin{bmatrix} v_1 \\ v_2 \\ v_3 \\ v_{4/arm} \\ v_{4/1} \\ v_{4/2} \\ v_{5/f} \\ v_{5/b} \\ v_6 \\ e_1 \\ e_2 \\ e_3 \\ e_4 \end{bmatrix} = \begin{bmatrix} 0 \\ 0 \\ 0 \\ 0 \\ 0 \\ 0 \\ 0 \\ 0 \\ 0 \end{bmatrix}$$

This equation, of the same format as equation S1, together with the previously shown lower and upper bounds for all 13 fluxes, can be used in any constrained-based approach for predicting phenotype. Finally, note that in this representation the equivalent of the original stoichiometric matrix is maintained in the upper-left corner of the matrix (see the highlighted submatrix).

1.5. Going genome-scale

In this study we applied the previously described framework to the latest version of the consensus genome-scale model of yeast[3]. Figure 1 in the manuscript shows a summary of the approach for constructing the new S matrix. It is a generalization of the approach used in the toy model example, but for a model that has n reactions and m metabolites, and in which p enzymes will be introduced. Note that the yellow lines divide the new stoichiometric matrix in 4 submatrices: the upper left submatrix is the original stoichiometric matrix (but modified to account for only irreversible reactions with no isozymes, as described previously), the upper right submatrix has only zeros, the lower left submatrix has the kinetic information and the lower right submatrix is an identity matrix.

It is important to mention that the lower left submatrix is not a diagonal matrix; as shown in section 0 it can have several coefficients in the same row (i.e. promiscuous enzyme) or column (i.e. complex), representing the substrate specific enzyme activities.

It is also relevant to notice that as any other stoichiometric matrix, the columns indicate each reaction's stoichiometry and the rows indicate mass balances for each metabolite (or enzyme). In particular, combining the enzyme usage's upper bound and its mass balance we can arrive to equation S3, which was the goal of this approach.

We have shown the mathematical basis for our approach; we will proceed therefore in the next section to detail how was this approach implemented.

2. Description of the method

The GECKO toolbox (**G**enome-scale model enhancement with **E**nzymatic **C**onstraints, accounting for **K**inetic and **O**mics data) can be found in the GitHub repository https://github.com/SysBioChalmers/GECKO/releases/tag/v1.0. Its main function is to transform a genome-scale model to account for enzymatic constraints. It is mainly written in MATLAB with a small section written in Python (for querying the BRENDA database). For running it, the only thing that should be done (beside updating the data from BRENDA, SWISS-PROT and KEGG) is run the function *enhanceGEM.m*, which has as an input the genome-scale model, the used toolbox (COBRA[4] or RAVEN[5]) and the new name for the model. In the following we will review how GECKO works, by 1) describing how k_{cat} values and other enzyme data was collected, 2) how the model is processed to a format suited to apply our approach, 3) how k_{cat} values are matched to the corresponding enzymes/reactions 4) how the enzymes are added to the model, and 5) how enzyme levels can be constrained in the model.

2.1. Retrieving enzyme data

2.1.1. Retrieving k_{cat} values: querying BRENDA

The BRaunschweig ENzyme DAtabase (BRENDA)[6] gathers enzymatic information, and can be queried by enzyme commission (EC) number and organism. A small package for automatically querying it was developed using Python as the scripting language, which can be found inside the GECKO toolbox. The following scripts should be ran (in the presented order) to obtain files with all enzymatic data:

1. ***retrieveBRENDA.py*:** Access the web client and retrieves all EC data from BRENDA. Creates files with the raw BRENDA output for each EC number for which there is data, with the file name '*ECX.X.X.X_FEATURE.txt*', where X.X.X.X is the EC number and FEATURE is one of the following 6: KCAT (k_{cat} values), KM (k_m values), MW (molecular weights), PATH (associated metabolic pathways), SEQ (sequences) or SA (specific activities. Note that each files contains the available information for all organisms.

2. ***createECfiles.py*:** Reads the previously generated raw files and creates easy to read EC files, with the file name '*ECX.X.X.X.txt*', where X.X.X.X is the EC number. These files have 5 columns: feature (one of the 6 previously mentioned), organism, value (value of the feature), substrate (when relevant) and comments (if any). Missing information is replaced with a *. Note that only the KCAT field is used onwards by the method, but the other fields are anyways added for manually checking consistency.

3. ***findMaxKcats.py*:** Reads the previously generated EC files and finds the maximum k_{cat} for each substrate for a chosen microorganism. Writes a table with the following columns (from left to right): EC number, substrate, maximum k_{cat} value for the organism (s^{-1}), k_{cat} standard deviation for the organism (in case of several measurements), maximum k_{cat} value for the rest of the organisms (s^{-1}), and k_{cat} standard deviation for the rest of the organisms. The generated table is stored in a file named '*ORGANISM_max_kCATs.txt*', where ORGANISM is the name of the organism chosen. As a general rule the maximum k_{cat} value (i.e. the highest turnover rate) was chosen to avoid over-constraining the model later on[7].

All results shown in this study were attained using the k_{cat} values gathered with these scripts (ran on August 26th 2015), choosing *S. cerevisiae* as the organism in *findMaxKcats.py*.

2.1.2. Retrieving other enzyme data: querying SWISS-PROT and KEGG

All other enzyme data besides the k_{cat} values was retrieved from SWISS-PROT[8] and KEGG[9]. Both databases were manually accessed online (SWISS-PROT on April 30th 2015, KEGG on April 30th 2014), and all available information for *S. cerevisiae* was downloaded as text files. The script *updateDatabases.m* (available in the GECKO toolbox) processes these files, and creates a file named '*ProtDatabase.mat*', which contains 2 relevant structures:

- **swissprot:** A table with 6 columns: UNIPROT code of protein, protein name, associated genes, associated EC numbers (if any), molecular weight of protein (calculated from the sequence using *calculateMW.m*) and full sequence.

- **kegg:** A table with 7 columns: UNIPROT code of protein, protein name, associated genes, associated EC numbers (if any), molecular weight of protein (calculated from the sequence using *calculateMW.m*), associated pathways (if any) and full sequence.

2.2. Genome-scale model pre-processing

The latest version of the consensus genome-scale model of yeast[3] was used in this work. The downloaded release from the project's repository (https://sourceforge.net/projects/yeast/) was Yeast 7.6, on July 9th 2015. Before adding the enzymes to the model, a series of modifications were performed, which are detailed in the following.

2.2.1. Model corrections

The script *modelCorrections.m* (available in the GECKO toolbox) contains a set of modifications made to the model to fix some issues. Namely:

- **Correct glucan coefficients in biomass pseudo-reaction:**

Checking the biomass composition of the model we noticed that the total fraction of carbohydrates added up to 0.59 g/gDW (Table S1), which is an 45% overestimation of the literature value of 0.41 g/gDW[10]. Specifically, we found that in the downloaded model there are two types of glucan being used as components in the biomass pseudo-reaction (reaction code *r_4041* in the model): (1->3)-beta-D-glucan in the cytoplasm (metabolite code *s_0002* in the model) and (1->6)-beta-D-glucan in the cell envelope (code *s_0004*). Even though both of these components should add up to 1.135 mmol/gDW together[10], each of them had a value of 1.135 mmol/gDW, hence doubling the amount of glucan and creating the aforementioned error (Table S1). To correct this issue we set at zero the composition of (1->3)-beta-D-glucan in the cytoplasm and distributed the 1.135 mmol/gDW of (1->6)-beta-D-glucan in the cell envelope together with (1->3)-beta-D-glucan also in the cell envelope (*s_0001*), in a proportion of 25% - 75% respectively[11]. With these modifications we achieved the correct carbohydrate composition of 0.41 g/gDW in yeast (Table S1).

Table S1: Original and corrected coefficients in the carbohydrate composition of the biomass pseudo-reaction of Yeast 7.

Metabolite name in model	Metabolite code	Molecular weight [g/mol]	Composition [mmol/gDW]		Composition [g/gDW]	
			Original	Corrected	Original	Corrected
(1->3)-beta-D-glucan [cytoplasm]	s_0002	180.2	**1.1348**	**0**	**0.184**	**0**
(1->3)-beta-D-glucan [cell envelope]	s_0001	180.2	**0**	**0.8506**	**0**	**0.138**
(1->6)-beta-D-glucan [cell envelope]	s_0004	180.2	**1.1348**	**0.2842**	**0.184**	**0.046**
chitin [cytoplasm]	s_0509	221.2	1E-6	1E-6	2.03E-7	2.03E-7
glycogen [cytoplasm]	s_0773	180.2	0.5185	0.5185	0.084	0.084
mannan [cytoplasm]	s_1107	180.2	0.8079	0.8079	0.131	0.131
trehalose [cytoplasm]	s_1520	342.3	0.0234	0.0234	7.59E-3	7.59E-3
**TOTAL**	-	-	-	-	**0.59**	**0.41**

- **Correct extracellular membrane potential:**

  When preliminary testing the model's capabilities, we realized that protons could be exported out of the cell using a transport (*r_1824*) with no ATP cost. This is inaccurate; the extracellular pH is usually orders of magnitude higher than the intracellular pH, so proton diffusion is very unlikely to occur from the inside out, and instead it typically occurs with the help of the cytoplasmic ATPase, representing therefore a significant energy expenditure associated to yeast growth. In order to fix this problem, we blocked the reversibility of this transport, allowing it only to uptake protons freely, and also blocked the free export of putrescine and spermidine (*r_1250* and *r_1259*, respectively), in order to avoid together with putrescine/H^+ and spermidine/H^+ antiporters unfeasible loops that were still allowing free excretion of protons. After these 3 modifications proton excretion had an associated ATP cost as expected.

- **Correct coefficients in oxidative phosphorylation:**

  Three main changes were made in the model's oxidative phosphorylation pathway:

  1. The proton pumping of complexes III (*r_0439*) and IV (*r_0438*) was assumed to have a 63.3% efficiency. This was done to represent a 63.3% efficiency of the

mechanistic value of the P/O ratio, value observed in experimental data of yeast growing on glucose[12].

2. The stoichiometry in complex IV was normalized by the number of ferrocytochromes c (*s_0710*), to have a correct stoichiometry enzyme to ferrocytochrome.
3. The stoichiometry of ATP synthase (complex V; *r_0226*) was changed to correctly represent the ratio H^+/ATP of 3/1 common in yeast, not the original 4/1 which applies to organisms that have complex I present[13].

- **Delete blocked reactions:**

  4 reactions that had upper and lower bound equal to zero were removed: putrescine and spermidine excretions (*r_1250* and *r_1259*, respectively), and previous biomass pseudo-reactions for models yeast 5 and yeast 6 (*r_2110* and *r_2133*, respectively).

- **Correct reversibility vector:**

  The model's field *model.rev* was corrected to indicate whether a reaction is reversible (can carry both positive and negative flux) or irreversible (can only carry positive flux). An exception was made for exchange reactions: they remained as reversible even if they could only carry positive flux, in order to have the possibility to supply the model with different media.

- **Remove unused field:**

  Some fields of the model were removed, whether because they were empty or not relevant for our analysis: *model.metCharge*, *model.subSystems*, *model.confidenceScores*, *model.rxnReferences*, *model.rxnECNumbers*, *model.Notes*, *model.metChEBIID*, *model.KEGGID*, *model.metPubChemID* and *model.metInChIString*.

2.2.2. Standardization of model

In order to have an easier to visualize model in posterior analysis, some additional changes were performed, mainly including additional fields with information about the compartment of each

metabolite. Those are detailed in *standardizeModel.m* (available in the GECKO toolbox) and in the following:

- Removal of any compartment reference in the field *model.MetNames*; if a metabolite is called '*metabolite_X [compartment]*' then the name is replaced by '*metabolite_X*' and the compartment stored elsewhere.
- Creation of an additional field named *model.metComps*, which is a numerical vector that indicates in which compartment each metabolite is.
- Creation of an additional field named *model.compNames*, which is a cell array that denotes the full name of each compartment. The numbers in *model.metComps* correspond to the position of the compartment in this field.
- Creation of an additional field named *model.comps*, which is of the same size of *model.compNames* and contains abbreviated names for each compartment.

2.3. Matching k_{cat} data to the model

Before adding enzymes to the model, we need to first find which enzyme (or groups of enzymes) catalyze(s) each reaction, and for each pair enzyme/reaction find an appropriate k_{cat} value. This is done using the databases created previously (see sections 2.1 and 2.1.2) and is detailed in the following.

2.3.1. Matching reactions to enzymes

The first necessary step is, for each reaction, to find the enzymes (or complexes) that can catalyze it in the SWISS-PROT or KEGG constructed databases (section 2.1.2). This is attained with the following functions (all available in the GECKO toolbox):

- ***getEnzymeCodes.m*:** Main loop that goes through all reactions of the model. First will try a match in SWISS-PROT, and if not found will try a match in KEGG. Returns a matrix with the associated proteins (both uniprot codes and EC numbers) for each reaction as rows. If isozymes are present they will be shown in different columns. Also returns the substrates for each reaction (and the products if the reaction is reversible), for later k_{cat} matching in BRENDA.

- ***getAllPath.m*****:** Simplifies a given gene-reaction rule in terms of the isozymes. Receives a rule and returns the decomposed rule as an array, in which each row will have one possible isozyme. This can be done for single enzymes but also if complexes are also present. As an example, consider the rule $\mathrm{RR_j}$:

$$\mathrm{RR_j}:\ \ (\mathrm{G_1\ OR\ G_2})\ \mathrm{AND}\ (\mathrm{G_3\ OR\ G_4})$$

  These point out that there are 4 possible "isozyme" combinations, and those 4 combinations will be returned by this function:

$$\mathrm{RR_{j-1}}:\ \ \mathrm{G_1\ AND\ G_3}$$

$$\mathrm{RR_{j-2}}:\ \ \mathrm{G_1\ AND\ G_4}$$

$$\mathrm{RR_{j-3}}:\ \ \mathrm{G_2\ AND\ G_3}$$

$$\mathrm{RR_{j-4}}:\ \ \mathrm{G_2\ AND\ G_4}$$

- ***findInDB.m*****:** Matches each specific reaction gene rule to proteins in a database (SWISS-PROT or KEGG). First uses *getAllPath.m* to decompose the rule into different combinations, and then for each combination will try to find a match in the database for all genes. In the case of complexes, it will later check if there is an intersection between all matches and if so will return that one as associated protein. If not, it will return the union of all. The information that this function returns is the UNIPROT code, the EC number and the molecular weight.

2.3.2. Automatic k_{cat} matching criteria

Once we have all enzyme associations to each reaction, we attempt to match each protein/reaction pair to a k_{cat} measurement in BRENDA. This is done by the script *matchKcats.m* (available in the GECKO toolbox), which for each reaction (in both directionalities in the case of reversible reactions) will attempt a match, based on the following criteria:

- As a first option, it will try to match the EC number, the organism (in this case *S. cerevisiae*) and the corresponding substrate to some k_{cat} annotation in the BRENDA database.
- If no match is found, it will try to match the EC number and the substrate, but with any organism available.
- If still no match is found, it will try to match the EC number and the organism, but with any substrate available.
- If still no match is found, it will just try to match the EC number, with any substrate or organism available.
- If still no match is found, then it will introduce one wildcard to the EC number and attempt all previous 4 steps again. For example, if for the EC number EC1.2.3.4 no match was found with the previous 4 options, it will repeat all 4 options in the same order but for EC1.2.3.X (meaning that any EC number starting with EC1.2.3 will be considered a match). If still no match is found, it will repeat all 4 options with EC1.2.X.X and so on, until a match is found.

In all cases, if more than one match is found, the maximum value of all matches will be used (following the same consistence as when querying the BRENDA database).

2.3.3. Further manual curation of enzyme data

After running preliminary simulations it was clear that additional manual curation on the retrieved values was needed. For central carbon metabolism enzymes we replaced all values with previously manually curated data[7]. Additional changes are detailed in the following; most of them were annotation problems or lack of measurements. Each time specific activity was used, it was correspondingly multiplied by the molecular weight, to have the appropriate units (given that $\mathrm{k_{cat}} = \mathrm{s.a.} \times \mathrm{M.W.}$).

- **Aconitase (P19414/EC4.2.1.3):** The associated reactions in the model are represented as two-step reactions (*r_0280* and *r_0302* in the mitochondria, *r_0303* and *r_2305* in the cytoplasm), so each of the 4 k_{cat} values was multiplied by 2.
- **Fatty Acid Synthase (P07149+P19097/EC2.3.1.86):** No k_{cat} value was available in BRENDA, so instead *S. cerevisiae*'s FAS maximum specific activity for NADPH in BRENDA was used (3 μmol/min/mg [14]), divided by the stoichiometry of NADPH in the associated reactions in Yeast 7.6; 0.214 μmol/min/mg was used for producing palmitoyl-

CoA (14 NADPH molecules in *r_2140*) and 0.188 µmol/min/mg for producing stearoyl-CoA (16 NADPH molecules in *r_2141*).

- **Glycogen Synthase (P27472/EC2.4.1.11):** No k_{cat} value was available in BRENDA, so the value was corrected using 90.5 µmol/min/mg, *S. cerevisiae*'s glycogen synthase maximum specific activity for glucose (*r_0510*) in BRENDA [15].
- **Ketol-acid Reductoisomerase (P06168/EC1.1.1.86):** This enzyme participates in two pathways in the model: the biosynthesis of L-valine (reaction *r_0096*) and L-isoleucine (reaction *r_0669*). In the former reaction, the substrate had a different identifier in BRENDA (2-acetolactate) and in the model (2-acetyllactic acid), therefore no substrate match was found by the algorithm. Hence, the k_{cat} value was manually changed to 18.3 s^{-1} [16]. The same happened in the latter reaction; the identifier in BRENDA was 2-aceto-2-hydroxybutyrate but in the model was (S)-2-acetyl-2-hydroxybutanoate. The value was therefore also changed manually, to 78.3 s^{-1} [16].
- **Phosphoribosylformylglycinamidine synthase (P38972/EC6.3.5.3):** The only k_{cat} value available in BRENDA was for NH_4 (not a substrate in reaction *r_0079*) in *Escherichia coli*. The value was therefore corrected using the enzyme's maximum specific activity available for any organism (the measurement for *S. cerevisiae* was not available), equal to 2 µmol/min/mg [17].
- **HMG-CoA reductase (P12683-P12684/EC1.1.1.34):** The only k_{cat} value available in BRENDA was for *Rattus Norvegicus*, so the value was corrected using 0.027 µmol/min/mg, *S. cerevisiae*'s specific activity of the enzyme (reaction *r_0558*)[18].
- **FPP synthase (P08524/EC2.5.1.1):** The EC number is out of use; the recommended one is instead 2.5.1.10. The k_{cat} value was therefore corrected using 2.33 µmol/min/mg, *S. cerevisiae*'s specific activity of the enzyme (reactions *r_0355* and *r_0462*) [19].
- **Amino-acid N-acetyltransferase (P40360-Q04728/EC2.3.1.1):** The only k_{cat} value available in BRENDA was for *Mycobacterium tuberculosis*. The value was therefore corrected using the specific activity in *E. coli* from BRENDA, equal to 133 µmol/min/mg [20] (this was done for both isozymes in reaction *r_0761*).
- **Glutamate N-acetyltransferase (Q04728/EC2.3.1.1):** The EC number was missannotated (should be 2.3.1.35), but then no k_{cat} values were found for the correct EC number. The

value was hence corrected with the specific activity for *S. cerevisiae* in BRENDA, equal to 22 μmol/min/mg [21] (reaction *r_0818*).

- **α,α-trehalase (P32356/EC3.2.1.28):** The available k_{cat} values were not for *S. cerevisiae*. The value was hence corrected with the specific activity for *S. cerevisiae*, equal to 22 μmol/min/mg [22] (reaction *r_0194*).
- **Ribose-phosphate pyrophosphokinase (Q12265/EC2.7.6.1):** The substrate had a different identifier in BRENDA (D-ribose 5-phosphate) than in the model (ribose-5-phosphate), so it was using instead ATP as substrate (100 times lower). Hence, the k_{cat} value was manually changed to 60.68 s^{-1} [23] (reaction *r_0916*).
- **Glutamine synthetase (P32288/EC6.3.1.2):** No data was available in BRENDA for yeast or fungi. A manual search yielded a value of 236 μmol/min/mg for the specific activity in *S. cerevisiae* [24] (reaction *r_0476*).
- **Chorismate synthase (P28777/EC4.2.3.5):** No k_{cat} values available in BRENDA for *S. cerevisiae*. The value was therefore corrected using the specific activity in *E. coli* from BRENDA, equal to 14.8 μmol/min/mg[25] (reaction *r_0279*).
- **Homoaconitase, mitochondrial (P49367/EC4.2.1.36):** No k_{cat} values were available in BRENDA for *S. cerevisiae* (reactions *r_0027* and *r_0542*). We therefore used aconitase's k_{cat} value (143.3 s^{-1} [7] multiplied by two, as mentioned previously) together with a study in yeast[26] that shows that aconitase (P19414/EC4.2.1.3) is 0.062/0.005 = 12.4 times faster than homo-aconitase (0.062 and 0.005 are the specific activities of aconitase and homo-aconitase, respectively, measured in ΔOD/min/mg at 0-30% saturation with their corresponding substrates).
- **Formyltetrahydrofolate synthetase (P07245/EC6.3.4.3):** The k_{cat} value for *S. cerevisiae* in BRENDA was hidden as 'additional information'. The value was manually checked to be 200 s^{-1} [27] (reaction *r_0446*).
- **Methenyltetrahydrofolate cyclohydrolase (P07245/EC6.3.4.3):** The EC number was missannotated (should be 3.5.4.9). The value was hence corrected with the only wildtype k_{cat} value available in BRENDA, equal to 134 s^{-1} [28] (reaction *r_0725*).
- **Methylenetetrahydrofolate dehydrogenase (P07245/EC6.3.4.3):** The EC number was missannotated (should be 1.5.1.5), but then no k_{cat} values were found for the correct EC

number for *S. cerevisiae*. The value was correspondingly corrected using the specific activity for *S. cerevisiae* in BRENDA, equal to 259 µmol/min/mg[29] (reaction *r_0732*).

- **Phosphoserine transaminase (P33330/EC2.6.1.52):** The only k_{cat} values in BRENDA were for *E. coli* using fusion proteins. The value was hence changed using a specific activity found with a manual search[30], equal to 78 µmol/min/mg (reaction *r_0918*).
- **Succinate-semialdehyde dehydrogenase (P38067/EC1.2.1.16):** The retrieved k_{cat} value from BRENDA was from *E. coli* under extreme conditions. The value was corrected by using the specific activity for *S. cerevisiae* in BRENDA, equal to 0.66 µmol/min/mg[31] (reaction r*_1023*).
- **1,3-beta-glucan synthase component FKS1 (P38631/EC2.4.1.34):** The retrieved k_{cat} value from BRENDA was from *Staphylococcus aureus* and excessively low. The value was corrected by using the specific activity for *S. cerevisiae* in BRENDA, equal to 4 µmol/min/mg[32] (reaction *r_0005*).
- **Fructose-bisphosphate aldolase (P14540/EC4.1.2.13):** The protein was removed from missannotated reactions (*r_0322* and *r_0990*).
- **Golgi apyrase (P40009/ EC3.6.1.5):** The protein was removed from a missannotated reaction (r*_0227*).

Manual curation was performed to the pathway data as well, removing any KEGG classification for which there is no literature of its presence in yeast. These include pathway codes *sce00591* (linoleic acid metabolism), *sce00590* (arachidonic acid metabolism), *sce00592* (alpha-linolenic acid metabolism), *sce00565* (ether lipid metabolism), *sce00460* (cyanoamino acid metabolism) and *sce00680* (methane metabolism).

2.4. Adding enzymes to the model

After collecting all enzymatic data, pre-processing the genome-scale model and matching all reactions in the model with an associated enzyme to a corresponding k_{cat} value, the procedure can now create a new model which includes enzymes as metabolites, as described in section 1.3. This is done by scripts contained in the GECKO toolbox (the main call being to the function *readKcatData.m*), and consists of the following steps:

1. Converting the pre-processed model to an irreversible model, by using the RAVEN[5] adapted function *convertToIrreversibleModel.m* (available in the same folder as the rest of the functions). Reverse direction reactions are hence created named *r_XXX_REV*. All reactions are therefore converted to irreversible (i.e. LB = 0).
2. Going through all reactions with enzyme association (in both directions when possible) and one by one creating new reactions that include the enzymes as substrates. The main loop is done by the function *convertToEnzymeModel.m* and each addition is performed by *addEnzymesToRxn.m*.
3. In each new reaction creation, the only two things that are modified from the original reaction are the substrates (given that a new substrate, the enzyme, is added) and the corresponding stoichiometric coefficients in the S matrix (to match the inverse of the k_{cat} value, as previously shown). All other fields (LB, UB, obj, other stoichiometric coefficients) remain untouched. The reaction ID is redefined as *r_XXXNo1*.
4. In the case of isozymes, more than one reaction is created, each for a different enzyme, and therefore the IDs are redefined as *r_XXXNo1*, *r_XXXNo2*, etc. Additionally, an arm reaction named *arm_r_XXX* is created in these cases, as previously detailed (section 1.3)
5. In the case of promiscuous enzymes, the same enzyme is added in different reactions and no further action is needed.
6. In the case of complexes, several proteins are added at the same time to the new reaction. If no further manual data is known, the stoichiometry is assumed to be 1:1 in all cases.
7. After adding the new reaction(s), the original reaction *r_XXXX* is removed from the model.
8. After modifying all reactions in the model, all enzymes added to the new model's reactions (not counting repetitions) are collected in a single vector and for each one an enzyme usage pseudo-reaction is created, with the ID *prot_XXXXXX_exchange* (with XXXXXX being the UNIPROT number). This is done by the function *addProtein.m*.
9. Other fields in the model added by *addProtein.m* for each protein are: *model.enzymes* (containing the uniprot numbers from SWISS-PROT), *model.MWs* (containing the molecular weights calculated from the protein sequence), *model.sequences* (containing the protein sequences from SWISS-PROT), *model.genes2* (containing the gene associated to each enzyme from KEGG), *model.geneNames* (containing the common gene name from KEGG) and *model.pathways* (containing the KEGG associated pathways to the enzymes).

10. Finally, all previously mentioned manual curations performed in section 2.3.3 are included in the model, by the function *manualModifications.m*.

2.5. Constraining enzyme levels in the model

As a final step, we have included a module in the GECKO toolbox called *limit_proteins*, which has as main function *constrainEnzymes.m*. As input it requires the previously created model (section 2.4), the total measured protein content [g/gDW], an average saturation constant for unmeasured proteins, and an optional set of absolute protein abundances [mmol/gDW] with their corresponding UNIPROT IDs. It then performs the following transformations to the model:

1. Check all included enzymes in the model and define as upper bound of the corresponding enzyme usage the measured value (if the respective protein is part of the dataset).
2. Calculate $P_{measured}$, the aggregated mass of all matched enzymes, using the experimental values and the respective molecular weights.
3. Calculate f_m, the mass fraction of measured proteins in the model from the total:

$$f_m = \frac{P_{measured}}{P_{total}} \qquad [Eq. S22]$$

   Where P_{total} is the total amount of proteins, measured experimentally.

4. Calculate f, the mass fraction of unmeasured proteins in the model from all proteins not matched to the model (either because they were unmeasured or they were not part of the model to begin with):

$$f = \frac{f_n}{1 - f_m} \qquad [Eq. S23]$$

   Where f_n is the summed mass fraction of all unmeasured proteins included in the model, calculated with data from PaxDB[33] (Accessed April 7th 2015).

5. For all unmeasured enzymes still accounted in the model, create a constraint that represents the added sum of them, which should be less than the difference between P and $P_{measured}$, multiplied by f and a saturation factor (see section 2.5.1).

Note that in case of no proteomic data available, steps 1 and 2 will be skipped, f_m will be equal to zero, f will be equal to f_n, and the global constrain will be imposed on all enzymes. Some final modifications are also performed to the constrained model:

- The aminoacid composition in the model is scaled to reflect the total measured protein content by multiplying by f_P all aminoacid stoichiometric coefficients in the biomass pseudo-reaction (which were originally based on the biomass composition of chemostats at a dilution rate of 0.1 h^{-1} [10]):

$$f_P = \frac{P_{total}}{P_{base}} \qquad [Eq. S24]$$

Where P_{base} is the protein content at 0.1 h^{-1}, equal to 0.4005 g/gDW[10].

- To maintain an equivalent amount of mass in the biomass pseudo-reaction, it is assumed that the increase/decrease in aminoacid composition is compensated with a corresponding decrease/increase in the carbohydrate composition (something that is observed to a large extent experimentally[34]). Therefore all carbohydrate coefficients are multiplied by f_C:

$$f_C = \frac{C_{base} + P_{base} - P_{total}}{C_{base}} \qquad [Eq. S25]$$

Where C_{base} is the carbohydrate content in yeast at a dilution rate of 0.1 h^{-1}, equal to 0.4067 g/gDW[10].

- The growth associated maintenance (GAM), previously fitted for yeast to 59.276 mmol/gDW[10], comprises to a large extent polymerization costs, both for polymerizing aminoacids into proteins (16.965 mmol/gDW) and monosaccharides into polysaccharides (5.210 mmol/gDW). Because the composition of these two groups is changing in our model, the polymerization cost should change accordingly. Therefore, we fragment the model's GAM value into three amounts: one depending on the aminoacid composition, one depending on the carbohydrate composition and a third one to account for everything else, fitted manually for both aerobic and anaerobic conditions:

$$GAM_{total} = 16.965 \cdot P_{factor} + 5.210 \cdot C_{factor} + GAM_{fitted} \qquad [Eq. S26]$$

After the fitting procedure, GAM_{fitted} was equal to 31 mmol/gDW for aerobic conditions and 16 mmol/gDW for anaerobic conditions.

- Finally, the non-growth associated maintenance (NGAM) is set constant at 0.7 mmol/gDWh for aerobic conditions and 0 mmol/gDWh for anaerobic conditions.

2.5.1. In case of no protein data: the 'pool' assumption

For a large part of our study we had a lack of proteomic data, therefore instead of limiting each enzyme separately by its concentration, we limited the total amount of enzyme and let the model choose which amount of each enzyme should be used instead. As mentioned previously, this assumption is also used in case of partial proteomic data only with the unmeasured protein set. The following steps are performed:

1. Introducing an additional metabolite called *'prot_pool'*. This metabolite will represent an aggregated sum of all unmeasured enzymes present in the model.
2. Adding an usage pseudo-reaction for *prot_pool*:

$$\mathrm{ER_{pool}}: \ \rightarrow \mathrm{E_{pool}} \qquad [\mathrm{Eq. S27}]$$

   Note that this usage has units of protein mass per biomass dry weight [g/gDW].

3. Limiting this total usage with the unmeasured amount of protein:

$$\mathrm{e_{pool}} \leq (\mathrm{P_{total}} - \mathrm{P_{measured}}) \cdot \mathrm{f} \cdot \sigma \qquad [\mathrm{Eq. S28}]$$

   Here P_{total} is the total protein fraction in cell, which unless stated otherwise was assumed 0.4005 g/gDW[10], and $P_{measured}$ is the aggregated sum of measured proteins accounted in the model (equal to zero in case of no proteomic data). This difference is then multiplied by f, the mass fraction of proteins that are accounted in the model out of all proteins according to PaxDB[33] (Accessed April 7th 2015). In the case of no proteomic data, this value is equal to 0.4461 g/g for Yeast 7.6 (which indicates that 44.61% of yeast proteins mass-wise are included in our model). Finally, σ is a fitted parameter that represents the average saturation *in vivo* of enzymes.

4. Removing all other enzyme usage pseudo-reactions from the model and replacing them with a pseudo-reaction that draws from the enzyme pool towards each corresponding enzyme:

$$\mathrm{ER_i}: \ \mathrm{MW_i}\ \mathrm{E_{pool}} \rightarrow \mathrm{E_i} \qquad [\mathrm{Eq. S29}]$$

   Note that this pseudo-reaction "flux" has units of protein **amount** per biomass dry weight [mmol/gDW]. Also note that the stoichiometric coefficient for the substrate (the enzyme pool) is the molecular weight [kDa = g/mmol] of the corresponding enzyme; this is done so

because the enzyme pool should be distributed in terms of mass [g/gDW] but the reactions use enzymes molar-wise [mmol/gDW].

It is worthy to notice that the mass balance for any enzyme (Equation S11) does not change, with the exception that the flux e_i now refers to the transformation from *prot_pool* to enzyme E_i, instead of the usage of E_i. Even more interesting is the mass balance for *prot_pool* (obtained from equations S27 and S29):

$$e_{pool} - \sum_{i}^{P} MW_i \, e_i = 0 \qquad [Eq. S30]$$

Combining equations S28 and S30 and rearranging, we get:

$$\sum_{i}^{P} MW_i \, e_i \leq \sigma \cdot f \cdot (P_{total} - P_{measured}) \qquad [Eq. S31]$$

Which is conceptually equivalent to the approach known as metabolic modeling with enzyme kinetics (MOMENT)[35], an extension of FBA with molecular crowding (FBAwMC)[7,36], but limited to only unmeasured proteins. Our proposed framework is therefore flexible enough to constrain enzyme levels individually and/or constrain the total enzyme mass.

3. Additional methodology caveats

3.1. Visualization caveats

3.1.1. Classification of enzymes by metabolic group

Enzymes in the model were classified in metabolic groups in order to see differences in their distributions (Figures 2B and 2C in the manuscript). For achieving this, the pathway information available in KEGG was used, and KEGG pathways were classified in one out of three metabolic groups according to Table S2 (based on previous criteria[37]). Intermediate and secondary metabolisms were considered together as one metabolic group because there were too few enzymes belonging to secondary metabolism.

Table S2: KEGG pathways classified by three metabolic groups: Carbohydrate and energy primary metabolism (CE), aminoacid, fatty acid and nucleotide primary metabolism (AFN), and intermediate and secondary metabolism (IS).

Pathway	Classification
sce00010 Glycolysis / Gluconeogenesis	CE
sce00020 Citrate cycle (TCA cycle)	CE
sce00030 Pentose phosphate pathway	CE
sce00040 Pentose and glucuronate interconversions	CE
sce00051 Fructose and mannose metabolism	CE
sce00052 Galactose metabolism	IS
sce00061 Fatty acid biosynthesis	AFN
sce00062 Fatty acid elongation	AFN
sce00071 Fatty acid degradation	AFN
sce00072 Synthesis and degradation of ketone bodies	IS
sce00100 Steroid biosynthesis	IS
sce00130 Ubiquinone and other terpenoid-quinone biosynthesis	IS
sce00190 Oxidative phosphorylation	CE
sce00230 Purine metabolism	AFN
sce00240 Pyrimidine metabolism	AFN
sce00250 Alanine, aspartate and glutamate metabolism	AFN
sce00260 Glycine, serine and threonine metabolism	AFN
sce00270 Cysteine and methionine metabolism	AFN
sce00280 Valine, leucine and isoleucine degradation	AFN
sce00290 Valine, leucine and isoleucine biosynthesis	AFN
sce00300 Lysine biosynthesis	AFN
sce00310 Lysine degradation	AFN
sce00330 Arginine and proline metabolism	AFN
sce00340 Histidine metabolism	AFN
sce00350 Tyrosine metabolism	AFN
sce00360 Phenylalanine metabolism	AFN
sce00380 Tryptophan metabolism	AFN
sce00400 Phenylalanine, tyrosine and tryptophan biosynthesis	AFN
sce00410 beta-Alanine metabolism	IS
sce00430 Taurine and hypotaurine metabolism	IS

Table S2 (cont.): KEGG pathways classified by three metabolic groups: Carbohydrate and energy primary metabolism (CE), aminoacid, fatty acid and nucleotide primary metabolism (AFN), and intermediate and secondary metabolism (IS).

Pathway	Classification
sce00450 Selenocompound metabolism	IS
sce00480 Glutathione metabolism	IS
sce00500 Starch and sucrose metabolism	IS
sce00510 N-Glycan biosynthesis	IS
sce00513 Various types of N-glycan biosynthesis	IS
sce00514 Other types of O-glycan biosynthesis	IS
sce00520 Amino sugar and nucleotide sugar metabolism	IS
sce00561 Glycerolipid metabolism	IS
sce00562 Inositol phosphate metabolism	IS
sce00563 Glycosylphosphatidylinositol(GPI)-anchor biosynthesis	IS
sce00564 Glycerophospholipid metabolism	IS
sce00600 Sphingolipid metabolism	IS
sce00620 Pyruvate metabolism	CE
sce00630 Glyoxylate and dicarboxylate metabolism	CE
sce00640 Propanoate metabolism	IS
sce00650 Butanoate metabolism	IS
sce00670 One carbon pool by folate	IS
sce00730 Thiamine metabolism	IS
sce00740 Riboflavin metabolism	IS
sce00750 Vitamin B6 metabolism	IS
sce00760 Nicotinate and nicotinamide metabolism	IS
sce00770 Pantothenate and CoA biosynthesis	IS
sce00780 Biotin metabolism	IS
sce00790 Folate biosynthesis	IS
sce00860 Porphyrin and chlorophyll metabolism	CE
sce00900 Terpenoid backbone biosynthesis	IS
sce00909 Sesquiterpenoid and triterpenoid biosynthesis	IS
sce00910 Nitrogen metabolism	IS
sce00920 Sulfur metabolism	IS
sce00970 Aminoacyl-tRNA biosynthesis	IS

3.1.2. Connectivity of model

The metabolite network was constructed for both the original metabolic model and the enzyme-constrained model, in which nodes are metabolites and there is an edge between 2 nodes if they are present in the same reaction. The following connectivity metrics were computed for both the original metabolite network and the enzyme-constrained metabolite network:

- **Global clustering coefficient:** Scalar. Denotes the fraction of closed triplets in a network, in which a triplet is any three nodes sharing two connections and a closed triplet is a set of

three nodes sharing three connections. Represents how much the network clusters as a whole.

- **Local clustering coefficient (LCC):** Vector. For each node, denotes how well clustered is its vicinity (i.e. the fraction of connected nodes in its vicinity), in which a vicinity is all nodes that share a connection with the original node.
- **Average local clustering coefficient:** Scalar. The average of LCC.
- **Node degree (ND):** Vector. For each node, counts the amount of connections to other nodes. Represents how well connected is the specific node.
- **Average node degree (AND):** Scalar. The average of ND.
- **Shortest path matrix (SPM):** Matrix. For each pair of nodes, shows the length of the shortest path between those two nodes. The shortest path was computed with the Dijkstra algorithm[38].
- **Characteristic Path Length:** Scalar. The average of SPM.
- **Diameter (D):** Scalar. The highest value in SPM.
- **Path Diversity Matrix (SPD):** Matrix. For each pair of nodes, indicates the amount of paths that are equally short to the shortest path.
- **Average Path Diversity:** Scalar. The average of SPD.
- **Betweenness Centrality (BC):** Vector. For each node, denotes the average fraction of times that the node is present in shortest paths between all other nodes.
- **Average Betweenness Centrality:** Scalar. The average of BC.

All of these metrics were computed both with and without currency metabolites (which consisted of water, protons, carbon dioxide, oxygen, phosphate, diphosphate, ammonium, ATP, ADP, AMP, NAD(+), NADH, NADP(+) and NADPH) and are further detailed elsewhere[39].

3.2. Simulation caveats

3.2.1. Chemostat growth

Unless stated otherwise, the following procedure was followed to simulate chemostat growth:

1. Fix the specific growth rate at the dilution rate value (h^{-1}).
2. Remove constraints on any substrate uptake [mmol/gDWh].
3. Limit the total unmeasured enzyme mass [g/gDW] by 0.4005 g/gDW[10], f = 0.4461 g/g, and a saturation level of either $\sigma = 0.46$ for simulations of the CEN.PK113-7D strain (fitted to aerobic chemostats[40]) or $\sigma = 0.51$ for other strains (also fitted to aerobic chemostats[41]).
4. Minimize substrate uptake.
5. Fix substrate uptake to optimal value, allowing a 0.1% flexibility to avoid numerical errors.
6. Minimize enzyme usage, i.e. reaction ER_{pool} (equation S27). This to obtain the most efficient solution, similar to what is done in parsimonious FBA (pFBA)[42].

If proteomic data was available, then additionally each enzyme usage was limited with measured concentrations [mmol/gDW], and f was recalculated as mentioned in section 2.5.

Under chemostat conditions 5 exchange reactions were limited to physiological levels: production of pyruvate (reaction r*_2033*), acetate (reaction *r_1634*), (R,R)-2,3-butanediol (reaction *r_1549*), acetaldehyde (reaction *r_1631*) and glycine (reaction *r_1810*). The following criteria was followed:

- **CEN-PK strain**: Acetate was unconstrained and the other 4 exchange fluxes (unmeasured experimentally[40,43]) were limited (setting only the upper bound) by 1e-5 mmol/gDWh.
- **DS28911 strain**: Both acetate and pyruvate were limited by the maximum experimentally detected flux (0.62 mmol/gDWh and 0.05 mmol/gDWh, respectively[41]), and the other 4 exchange fluxes (unmeasured experimentally[41]) were limited by 1e-5 mmol/gDWh.

Additionally, in chemostat simulations, the L-serine transport between cytoplasm and mitochondria (reaction *r_2045*) was blocked to only operate forward, i.e. from the cytoplasm to the mitochondria, and the conversion of isocitrate to 2-oxoglutarate in the cytoplasm via NADPH (reaction *r_0659*) was blocked, as suggested previously for proper NADPH utilization[44].

3.2.2. Batch growth

Unless stated otherwise, the following procedure was followed to simulate batch growth:

1. Remove constraints on the corresponding carbon source uptake [mmol/gDWh].
2. Change the media accordingly to either minimal, with amino acids or complex, i.e. with amino acids and nucleotides. In the latter two cases, an upper bound of 2 mmol/gDWh was used for all exchange reactions.
3. Limit total unmeasured enzyme mass by 0.5 g/gDW, f = 0.4461 g/g, and a saturation level of $\sigma = 0.44$ (fitted to batch growth on glucose and minimal media[45]).
4. Maximize growth.
5. Fix growth at optimal value, allowing a 0.1% flexibility to avoid numerical errors. Minimize total sum of fluxes

In simulations of either fructose or mannose as sole carbon source, facilitated transport of the corresponding sugar was allowed, as low affinity transporters of glucose can equally accept fructose or mannose[46].

3.2.3. Flux variability analysis

The reduction in flux variability from the unconstrained model to the enzyme-constrained model was computed using Flux Variability Analysis (FVA)[47], both in the increasing dilution rate experiments under aerobic conditions (Section 2.3.1 in the manuscript) and in the integration of proteomic data (Section 2.4 in the manuscript). In the following we detail the procedure:

1. For each condition, the exchange fluxes of glucose, oxygen, CO_2, ethanol, acetate, glycerol and pyruvate, together with the dilution rate (growth), were fixed at the values predicted by the enzyme constrained model when minimizing for glucose at a fixed dilution rate, for both the original model and the enzyme-constrained model. To avoid numeric issues, a ±0.1% of flexibility was introduced to all mentioned exchange fluxes, i.e.:

$$\mathrm{LB} = 0.999 \cdot \mathrm{v_{pred-wMC}} \qquad [\mathrm{Eq.\,S32}]$$

$$\mathrm{UB} = 1.001 \cdot \mathrm{v_{pred-wMC}} \qquad [\mathrm{Eq.\,S33}]$$

2. Afterwards, for each reaction of each model 2 optimization problems were ran; one maximizing said reaction and one minimizing it. If the reaction was reversible, or had

several isozymes that could run it, the corresponding reactions were blocked, in order to avoid infinite loops. If any of the latter reactions turned essential for growth, then instead of blocked they were fixed at their natural value (taken from a minimization of glucose uptake with the constraints mentioned in step 1).

3. For each reaction, if the maximum flux predicted by the original model was more than a set threshold (1E-06 mmol/gDWh) over the minimum flux predicted by the original model, a reduction score was computed as follows:

$$\text{reduction} = \left(1 - \frac{\text{Max}_{\text{wMC}} - \text{Min}_{\text{wMC}}}{\text{Max}_{\text{ori}} - \text{Min}_{\text{ori}}}\right) \cdot 100\% \qquad [\text{Eq. S34}]$$

3.2.4. Random sampling

Both Yeast7 and ecYeast7 were simulated under random conditions in Section 2.4 of the manuscript in order to get a collection of flux distributions. For this a convex basis random sampling algorithm[48] was implemented for models in irreversible format:

1. The method starts by fixing the dilution rate of the model to 0.1 h^{-1} and the exchange fluxes of glucose, oxygen, CO_2, ethanol, acetate, glycerol and pyruvate to the values predicted by minimizing for glucose at the fixed dilution rate, accounting in the latter for a 10% variation.
2. Step 2 of the flux variability analysis shown in Section 3.2.3 is performed, to detect reactions that have a high variation (v_{max} - v_{min} > 999 mmol/gDWh). Those are not included in the random sampling, as they can take any value.
3. The random sampling is then performed in order to obtain 10,000 samples. Each sample is computed by the following procedure:
   - 3.1. Choose 3 fluxes in the network at random. The fluxes mentioned in step 2 are avoided in this random selection.
   - 3.2. Assign a random value between -1 and +1 (from a uniform distribution) to each of the chosen fluxes, and set this as a linear combination for the model's objective function.

3.3. If any of the 3 chosen reactions is reversible, or has several isozymes that can run it (in the case of the enzyme constrained model), the corresponding reactions are blocked, in order to avoid infinite loops.

3.4. Solve the optimization problem. If the problem turns unfeasible, i.e. at least one of the blocked reactions in step 3.3 is essential for growth, then a new optimization problem is run where instead of blocking those reactions they are fixed at their natural value (taken from the simulation performed in step 1).

3.5. Fix the values of the 3 chosen fluxes with the solution of the optimization problem, adding a ±0.1% of flexibility to avoid numerical issues.

3.6. Minimize the total flux sum in the network, to avoid loops among reversible reactions in the network. The solution of this final optimization problem is the sample saved for posterior analysis.

As shown in the final step of this method, this random sampling generates parsimonious samples, as all fluxes are minimized after fixing the chosen fluxes at the optimized values. This is unconventional in random sampling[48], however it is necessary given the irreversible format of the studied models.

3.2.5. Anaerobic growth in glucose-limited chemostats

In order to enforce anaerobic conditions in the model when needed, the following modifications were performed:

1. Oxygen uptake (*r_1992*) was blocked. Additionally, all reactions consuming oxygen (*s_1275* in the cytoplasm, *s_1276* in the endoplasmic reticulum, *s_1277* in the extracellular media, *s_1278* in the mitochondrion, *s_1279* in the peroxisome and *s_2817* in the endoplasmic reticulum membrane) were blocked as well.
2. The following exchange reactions were unblocked, in order to properly simulate anaerobic media (with fatty acids and sterols): ergosterol uptake (*r_1757*), lanosterol uptake (*r_1915*), zymosterol uptake (*r_2196*), 14-demethyllanosterol uptake (*r_2134*), ergosta-5,7,22,24(28)-tetraen-3beta-ol uptake (*r_2137*), oleate uptake (*r_2189*) and palmitoleate uptake (*r_1994*).

3. Heme a (*s_3714*) was removed from the biomass pseudo-reaction (*r_4041*), given that is needed for respiration, which is not active in anaerobic conditions, and 3 reactions involved in its synthesis require oxygen (*r_0304*, *r_0942* and *r_0530*).
4. Glycerol production should be present in anaerobic growth of yeast, acting as a NADH sink. For this to occur, the directionality of some reactions that were consuming NADH was fixed, including:
    - Malate dehydrogenase was fixed to not operate backwards. This was done both in the mitochondrion (*r_0713_REV*) and cytosol (*r_0714_REV*).
    - Glycerol dehydrogenase (*r_0487*) was fixed to not operate forward.
    - Glutamate synthase (*r_0472*) was also fixed to not operate forward.
5. Finally and as already stated in section 2.5, the growth associated ATP maintenance (GAM) was set to 16 mmol/gDW, and the non-growth associated ATP maintenance (NGAM) was set at 0 mmol/gDWh.

3.2.6. Proteomic integration

Absolute proteomic data was acquired from a recent study[49] of yeast growing aerobically in chemostats at 0.1 h^{-1} (triplicates), in the form of molecules/pgDW. The following transformations were sequentially applied to the proteomic dataset:

1. All measurements were transformed to mmol/gDW.
2. All zero values in the dataset were considered unmeasured data (NaN).
3. All enzyme entries that had 2 or 3 unmeasured values (NaN) were removed from the dataset.
4. For all remaining entries averages and standard deviations were calculated.
5. Each measurement was defined as the sum of the average and standard deviation, to allow flexibility in case of variable measurements. In total 0.025 g/gDW was included as this way.
6. Subunit measurements belonging to complexes II, III, IV and V in the model were forced to be proportional to their stoichiometry, given that some subunits were unmeasured, leading to an unfeasible model. In each case, as baseline for the proportions the subunit with the average relative abundance was chosen. In total 0.0016 g/gDW was added after these corrections.

After these transformations, the module *constrainEnzymes.m* was used to overlay protein measurements on the model (Section 2.5). Unmeasured enzymes were constrained using equation S31, with the following values: P_{total} = 0.448 g/gDW (according to experimental data[49]), $P_{measured}$ = 0.283 g/gDW (sum of all measured proteins in model, without the additional mass introduced for standard deviation and complex correction), f = 0.2154 g/g (fraction from $P_{total} - P_{measured}$ that is accounted in the model according to PaxDB) and σ = 0.46 (value optimized for CEN.PK113-7D, the strain used in this study).

3.2.7. Flux control coefficient analysis

Flux control coefficients (FCCs) were calculated as previously proposed[7], as a way of studying the effect on the simulation output of varying each enzyme's specific activity. For each enzyme, the corresponding k_{cat} value was increased in 0.1% and the model was simulated under batch conditions, to observe the percentage increase in the flux of interest v (specific farnesene production rate or specific growth rate). If a specific enzyme was promiscuous, the k_{cat} values of all associated reactions were increased by 0.1%. The FCC was defined as a relative sensitivity:

$$\mathrm{FCC_i} = \frac{\Delta v}{v_b} \Big/ \frac{\Delta k_{cat}^{ij}}{k_{cat}^{ij}} \qquad [\mathrm{Eq. S35}]$$

Which can be expanded to

$$\mathrm{FCC_i} = \frac{v_{up} - v_b}{v_b} \Big/ \frac{1.001 k_{cat}^{ij} - k_{cat}^{ij}}{k_{cat}^{ij}} \qquad [\mathrm{Eq. S36}]$$

And by simplifying we arrive to

$$\mathrm{FCC_i} = 1000 \frac{v_{up} - v_b}{v_b} \qquad [\mathrm{Eq. S37}]$$

Where v_b is the original flux value and v_{up} is the new flux value due to the change in the k_{cat} value.

4. Supplementary results

4.1. Description of the model

Table S3 shows how many k_{cat} values were obtained from different criteria by applying the GECKO algorithm to a GEM of *S. cerevisiae*[3]. Overall our method extracted 3249 values from BRENDA, from which more than 90% come from using at most 1 wild card, and more than 50% are values from *S. cerevisiae*. Figure S2 on the other hand shows histograms of complexes, isozymes and promiscuous enzymes. It can be seen that most complexes have 2 subunits (Figure S2A), most reactions that have isozymes have 2 (Figure S2B) and most promiscuous enzymes catalyze 2 types of reactions (Figure S2D). Finally, it is interesting to notice that according to the model, there are some promiscuous enzymes that are able to catalyze more than 20 and up to 192 different types of reactions (Figure S2C), something that is unlikely to happen *in vivo*. However, going through those reactions we saw that all of them are part of lipid metabolism, particularly of the synthesis of triglycerides, phospholipids and/or sphingolipids. Because these lipids have all 3 fatty acid chains, which can vary independently in length, Yeast 7.6 includes reactions for multiple combinations of them, and therefore the promiscuity of the associated enzymes is much higher.

Table S3: Number of k_{cat} values obtained by using different criteria with the GECKO toolbox.

	**No wild cards**	**1 wild card**	**2 wild cards**	**3 wild cards**	**4 wild cards**	**Total**
**Match found for substrate and *S. cerevisiae***	168	82	0	11	0	261
**Match found for substrate and any organism**	506	225	10	10	0	751
**Match found for any substrate and *S. cerevisiae***	110	1125	14	168	0	1417
**Match found for any substrate and any organism**	648	172	0	0	0	820
**Total**	1432	1604	24	189	0	3249

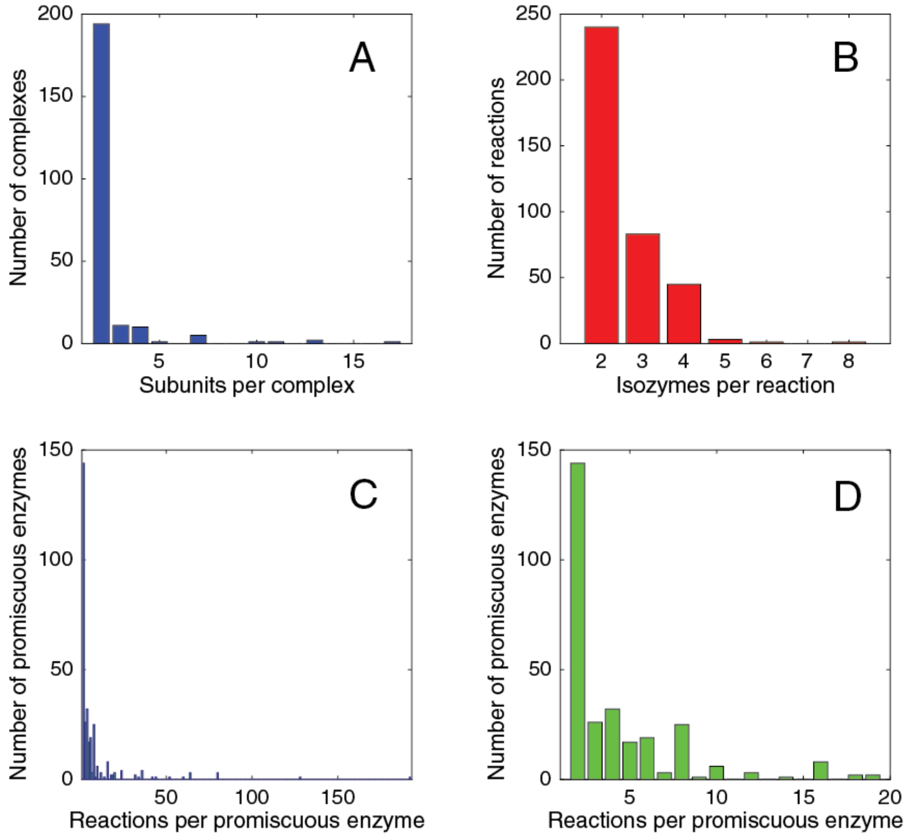

Figure S2: Histograms of the model. (A) Number of subunits per complex. (B) Number of isozymes per reaction with isozymes. (C) Number of reactions per promiscuous enzyme. (D) Number of reactions per promiscuous enzyme (zoom).

4.2. Enzyme features

Figure S3 displays how all 764 enzymes included in the model distribute in their molecular weights and average k_{cat} values. As expected[37], molecular weights (Figure S3A) have much less variation than k_{cat} values (Figure S3C) in terms of orders of magnitude; molecular weights span 3 orders of magnitude (the smallest being the plasma membrane ATPase proteolipid 1 with 4.5 kDa and the largest being acetyl-CoA carboxylase with 259.2 kDa), whereas k_{cat} values span 11 orders of magnitude (the slowest being serine/threonine-protein kinase with 1.6e-04 s^{-1} and the fastest being catalase T with 2.8e06 s^{-1}). Additionally, it is interesting to notice that there is no significant correlation between both variables (Figure S3B), i.e. between the size and speed of enzymes in our model.

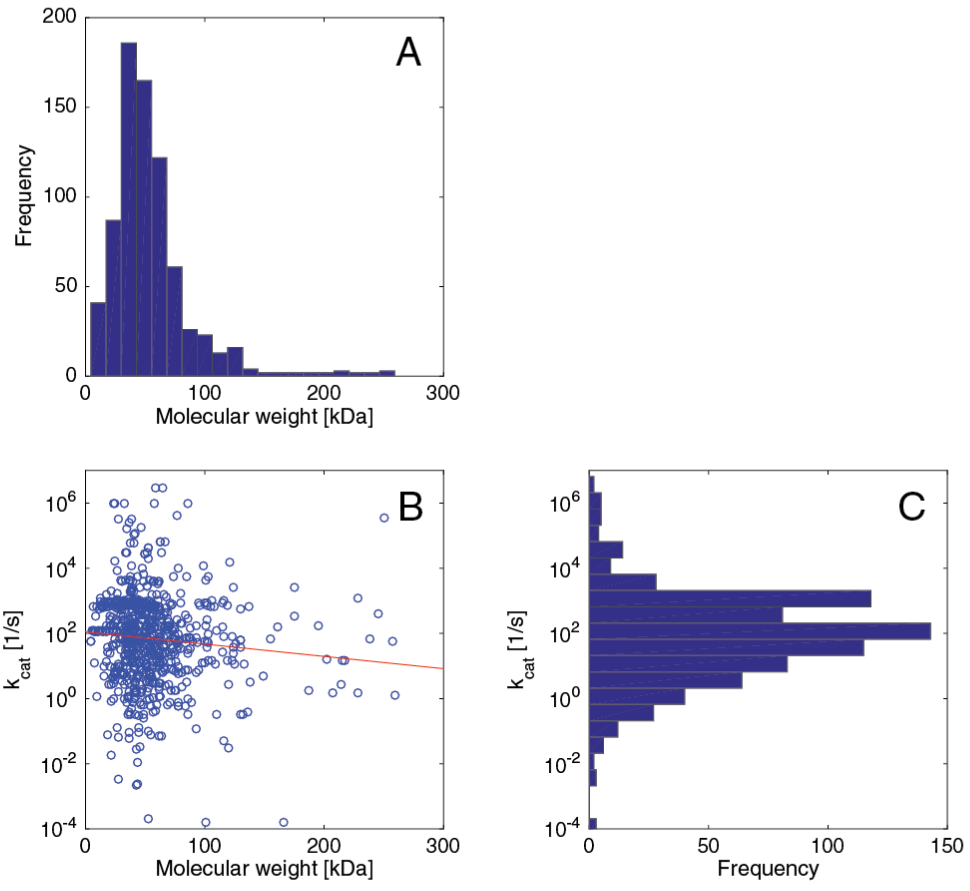

Figure S3: Overview of enzymatic data in model. (A) Histogram of enzymes' molecular weights. (B) Semi-logarithmic plot between enzymes' molecular weights and enzymes' k_{cat} values, showing that there is no strong linear correlation between both variables (red line, $R^2 = 0.010$). (C) Logarithmic histogram of enzymes' k_{cat} values.

Figures 2B and 2C in the manuscript show the cumulative distributions of k_{cat} values and molecular weights, respectively, of the model's enzymes separated by metabolic functions. All 3 groups are significantly different ($p < 0.05$ with a non-parametric Wilcoxon Rank-Sum test) both by k_{cat} value and molecular weight, as shown in Table S4.

Table S4: p-values across metabolic groups, for both k_{cat} values and molecular weights. CE = carbohydrate and energy primary metabolism. AFN = amino acid, fatty acid and nucleotide primary metabolism. IS = intermediate and secondary metabolism.

	**k_{cat} values**		**Molecular weights**	
	**AFN**	**IS**	**AFN**	**IS**
**CE**	0.01	1.6E-07	0.01	1.3E-08
**AFN**	-	0.03	-	4.7E-04

Figure S4A shows the cumulative distribution of average k_{cat} values for all model's enzymes, with a median value of 70.9 s^{-1}. Figure S4B compares proteins that are part of complexes (in total 173) versus proteins that operate as standalone enzymes (591). We see that the median of the former group (188.3 s^{-1}) is much higher ($p = 9.4e-08$ with a non-parametric Wilcoxon Rank-Sum test) than the median of the latter group (56.4 s^{-1}), suggesting that complexes tend to operate faster than single enzymes. Figure S4C shows enzymes catalyzing reactions with other isozymes (269) versus enzymes catalyzing reactions with no isozymes (495). In this case there is no significant difference ($p = 0.38$) between the median values (both of 70.9 s^{-1}), implying that reactions with isozymes or without isozymes tend to have similarly fast catalyzers. Finally, Figure S4D displays promiscuous enzymes (315) versus non-promiscuous enzymes (449), which also shows a significant difference ($p = 0.03$) between medians (100.0 s^{-1} Vs 70.9 s^{-1}, respectively), indicating that promiscuous enzymes tend to be faster than non-promiscuous enzymes.

Lastly, we can see the cumulative distribution of all molecular weights in Figure S5A, with a median value of 47.4 kDa. Also, we observe that proteins part of complexes (median MW = 39.7 kDa) are usually smaller ($p = 2.4e-07$) than standalone enzymes (median MW = 50.8 kDa) (Figure S5B), enzymes catalyzing reactions with isozymes (median MW = 53.9 kDa) are larger ($p = 1.8e-06$) than enzymes catalyzing reactions with no isozymes (median MW = 44.0 kDa) (Figure S5C), and that promiscuous enzymes (median MW = 50.8 kDa) are larger ($p = 0.02$) than non-promiscuous enzymes (median MW = 47.0 kDa) (Figure S5D).

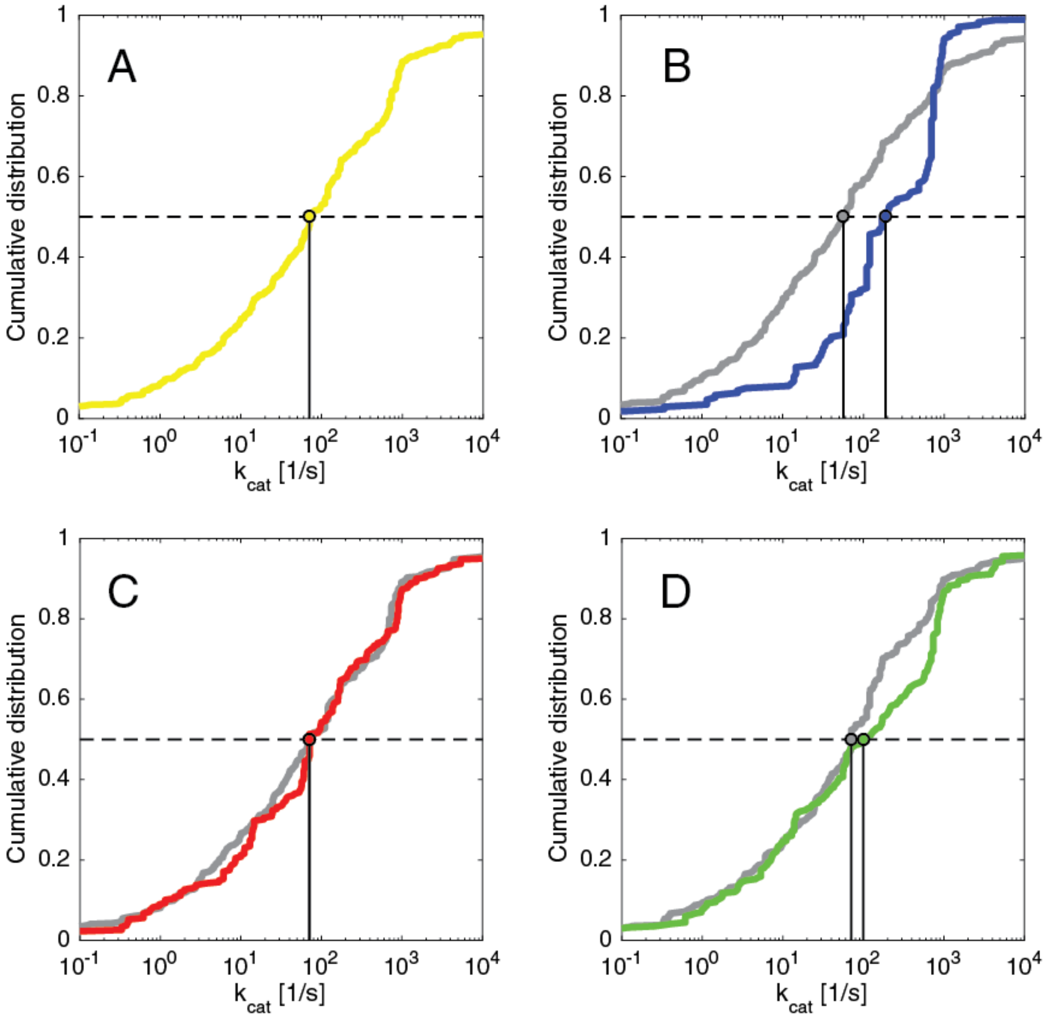

Figure S4: Cumulative distributions of k_{cat} values of the model's enzymes. Note that the scale is logarithmic. (A) All enzymes in model. (B) Proteins part of complexes (blue) Vs standalone enzymes (grey). (C) Enzymes catalyzing reactions with isozymes (red) Vs enzymes catalyzing reactions with no isozymes (grey). (D) Promiscuous enzymes (green) Vs non-promiscuous enzymes (grey).

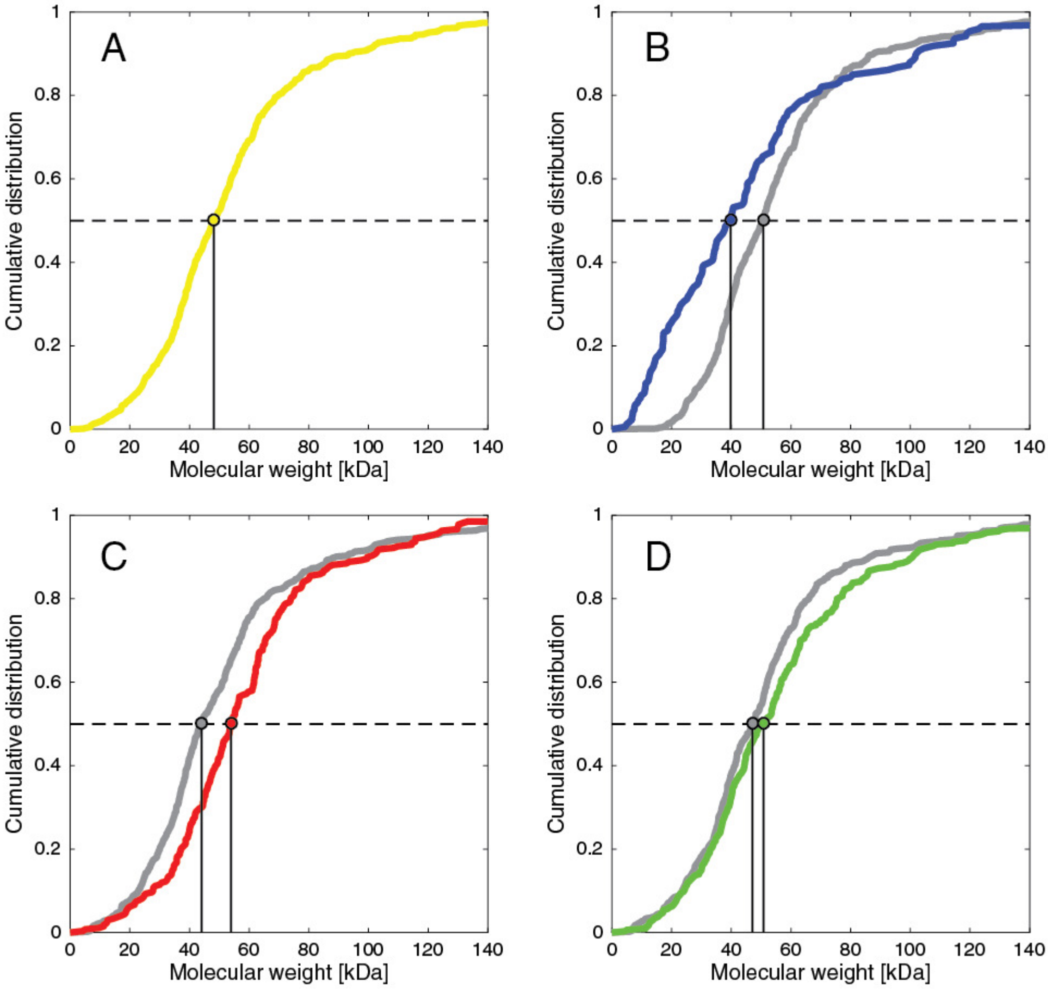

Figure S5: Cumulative distributions of molecular weights of the model's enzymes. (A) All enzymes in model. (B) Proteins part of complexes (blue) Vs standalone enzymes (grey). (C) Enzymes catalyzing reactions with isozymes (red) Vs enzymes catalyzing reactions with no isozymes (grey). (D) Promiscuous enzymes (green) Vs non-promiscuous enzymes (grey).

4.3. Connectivity of model

Figure S6 displays the node degree of the metabolite networks of both the original metabolic model and the enzyme-constrained model. It can be seen that the enzyme constrained model shows a higher node degree in general than the original model.

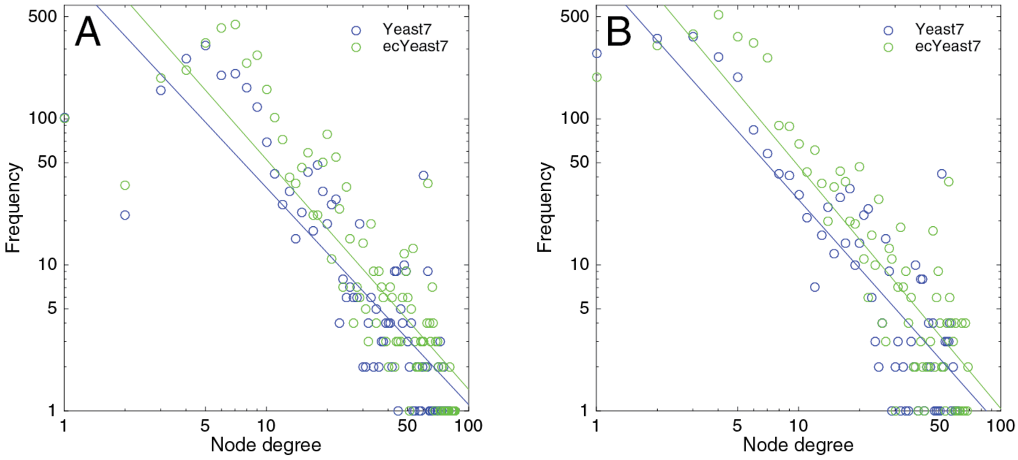

Figure S6: Node degree histograms of original metabolic model (blue) and enzyme-constrained model (green), (A) with and (B) without currency metabolites.

4.4. Chemostats with varying dilution rate

Figure S7 displays the flux predictions of the model compared to experimental data for both aerobic[41] and anaerobic[34] chemostats at different dilution rates. As expected, the original metabolic model performs poorly at high specific growth rate and fails to capture the overflow metabolism strategy (Figure S7A). On the other hand, the enzyme-constrained model shows enzyme limitation between the critical dilution rate of 0.3 h^{-1} and the maximum feasible dilution rate simulated of 0.45 h^{-1} (the latter not shown), and successfully predicts a Janusian region together with the production of ethanol and acetate (Figure S7B). A small Janusian region is observed as well at high growth rates under anaerobic conditions (Figure S7D), in which the ethanol production rate decreases and instead the glycerol production rate increases. This occurs between the dilution rates of 0.37 and 0.40 h^{-1}; once the latter dilution rate is reached the model is not able to grow anymore. None of the above mentioned behavior is captured by the original metabolic model, which instead predicts linear growth at all dilution rates (Figure S7C).

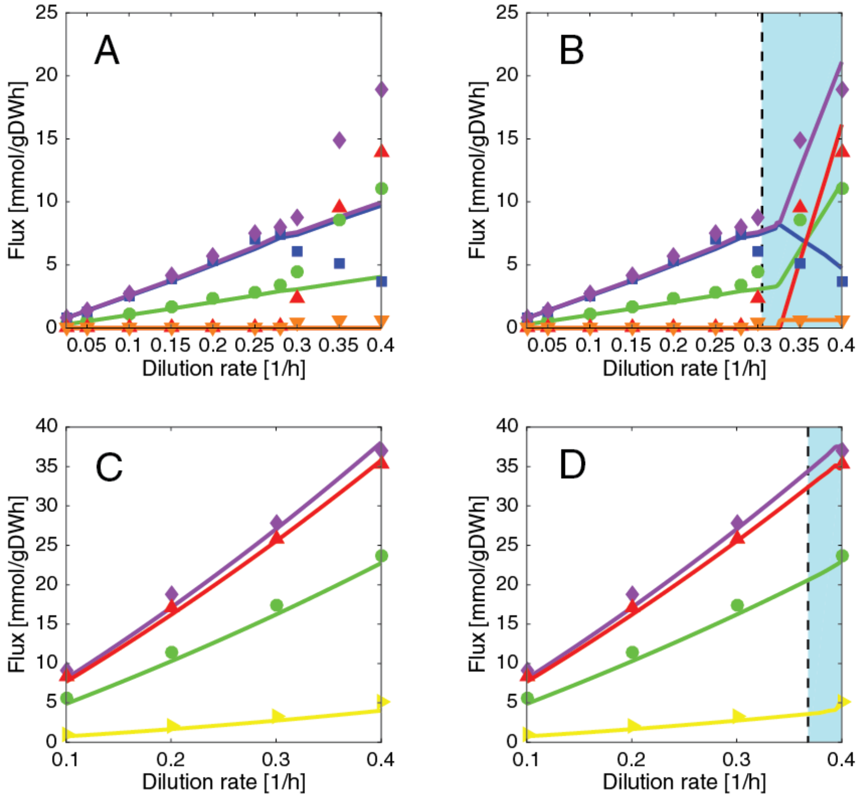

Figure S7: Model predictions (lines) and experimental values (points) for exchange fluxes from glucose-limited chemostats: glucose (green circles) and oxygen (blue squares) consumption, and CO_2 (purple diamonds), ethanol (red triangles), acetate (orange inverted triangles) and glycerol (yellow right-pointing triangles) production. (A) Yeast7; aerobic conditions. (B) ecYeast7; aerobic conditions (the light blue area denotes the region of both glucose and protein limitation). (C) Yeast7; anaerobic conditions. (D) ecYeast7; anaerobic conditions.

Figure S8 on the other hand shows 10,000 simulations of the enzyme-constrained model with randomly assigned k_{cat} values and 10,000 simulations with randomly assigned molecular weights, from gamma distributions fitted from the corresponding data. It can be seen that if either k_{cat} values or molecular weights are assigned randomly to the model, most of the times the model will not be able to predict the critical dilution rate (Figure S8B), the final oxygen consumption rate (Figure S8C) nor the final ethanol production rate (Figure S8D), overall fitting quite badly to the experimental data (Figure S8A).

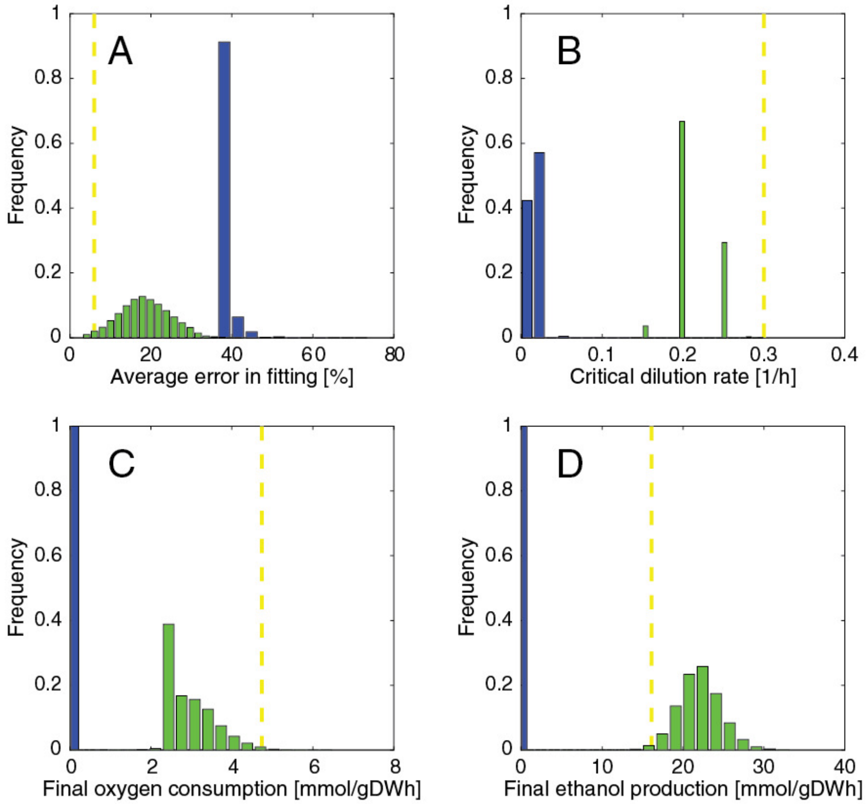

Figure S8: Performance of enzyme-constrained models with random k_{cat} values (blue) and molecular weights (green). In yellow the original selection for ecYeast7 is shown. (A) Overall fitting error to the experimental data (aerobic chemostats at increasing dilution rate[41]). (B) Dilution rate at which the protein content becomes limited. (C) Oxygen consumption rate at a dilution rate of 0.4 h^{-1}. (D) Ethanol production rate at a dilution rate of 0.4 h^{-1}.

Finally, Figure S9 shows the flux variability analysis (FVA) performed to both the original metabolic model Yeast7 (transformed to irreversible for a fair comparison) and the enzyme-constrained model ecYeast7, at a low growth rate (0.025 h^{-1}) and a high growth rate (0.4 h^{-1}). At low growth rates, 3286 reactions of Yeast7 have non-zero variability, which corresponds to 66.1% of the reactions of said model. In turn, ecYeast7 has 3958 reactions with non-zero variability, which is 58.7% of the model's reactions. Even though the medians are similar (Figure S9A), the distributions are significantly different (p = 1.7e-32 with a non-parametric Wilcoxon Rank-Sum test). At high growth rates on the other hand, 3304 reactions (66.5%) of Yeast7 have non-zero variability, which is higher than the 3847 reactions (57.1%) of ecYeast7. The distributions here are even further apart (Figure S9B), with a median ~34 times smaller and a significant difference among distributions (p = 1.5e-229). In conclusion, in both cases the enzyme-constrained model has considerably lower variability.

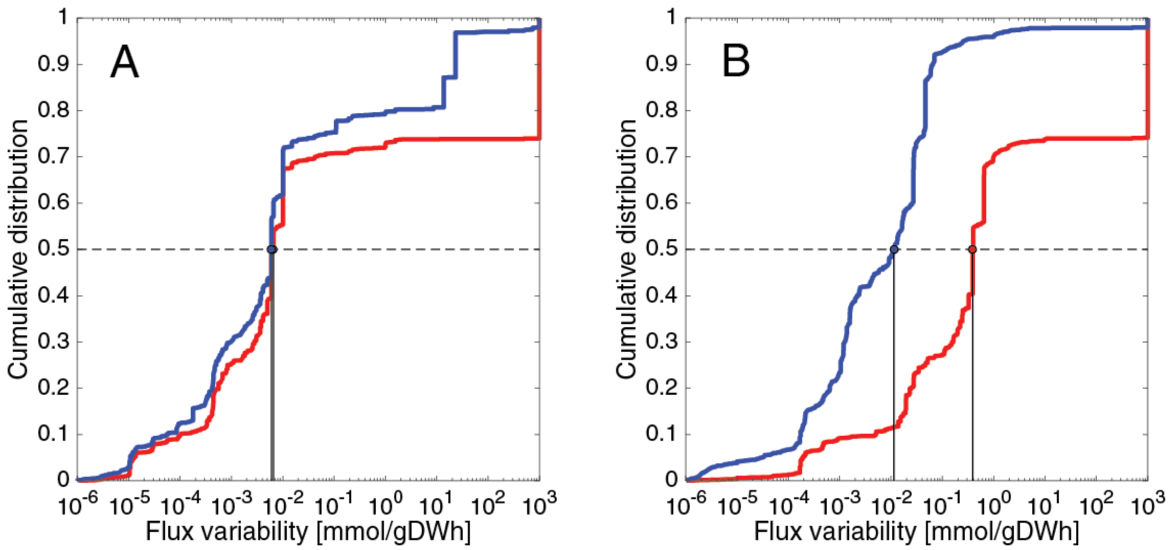

Figure S9: Cumulative distribution of fluxes with non-zero variability for Yeast7 (red) and ecYeast7 (blue), under (A) low specific growth rate = 0.025 h^{-1} and (B) high specific growth rate = 0.4 h^{-1}.

4.5. Growth under temperature stress

Figure S10 shows simulations of both Yeast7 and ecYeast7 with an unmodified NGAM = 0.7 mmol/gDWh. It can be seen that both models predict a very low glucose uptake and corresponding O_2 consumption and CO_2 production. Additionally, no ethanol production is observed. This points to the fact that under high temperature conditions additional maintenance energy is needed to cope with the stress, which causes the increase in glucose uptake.

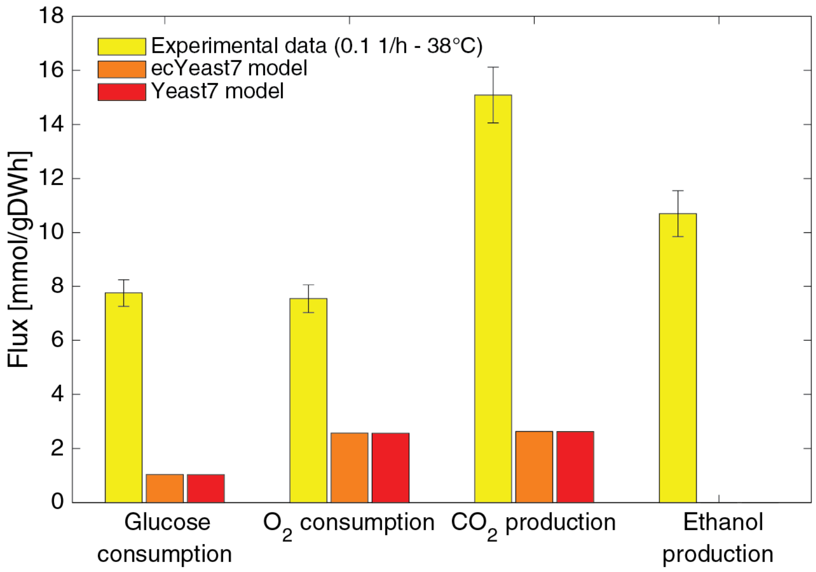

Figure S10: Both Yeast7 and ecYeast7 miss predict temperature stress experimental data when NGAM is unchanged.

4.6. Proteomic integration

Figure S11 displays the experimental exchange fluxes of both the purely metabolic model and the enzyme-constrained model under aerobic glucose-limited chemostat conditions, D = 0.1 h^{-1}. It can be seen that both models give similar predictions. Table S5 on the other hand shows, for both models as well, the internal distribution of some central carbon metabolism flux ratios, i.e. fluxes normalized by the glucose uptake. The predicted values are displayed together with experimental values obtained with ^{13}C metabolic flux analysis (^{13}C-MFA) from a previous study[50]. Even though for some reactions Yeast7 performs better and for some ecYeast7 does so, overall both models have similar predictive performance, as it can be seen in Figure S12.

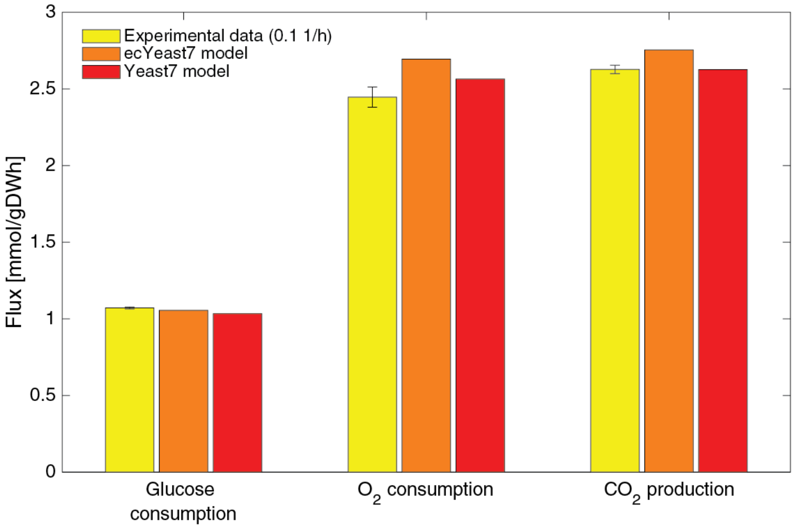

Figure S11: Flux predictions of the original metabolic model (Yeast7) and the enzyme constrained model (ecYeast7) compared to experimental data of yeast grown at 0.1 h^{-1} aerobically limited on glucose.

Table S5: Comparison of predicted flux ratios by Yeast7 and ecYeast7 to experimental values attained by ^{13}C-MFA from a previous study[50]. The predicted values were calculated by adding up the fluxes corresponding to the reaction sets shown in the 4th column. If a reaction had a reversible counterpart the corresponding flux was subtracted, except for the OAA transport (as both directions are present in the study). The metabolite sets G3P/3PG, Ru5P/Ro5P/X5P and OAA/MAL (the latter both in cytoplasm and mitochondria) were considered as one metabolite each, in order to adapt to the model proposed in the study with the experimental data[50].

Type of reaction	Reaction code (Jouthen et al. 2008)	Reaction(s)	Reactions matched from model	Average experimental flux ratio	Yeast7 flux ratio	ecYeast7 flux ratio
Cytoplasmic	x_1	glucose → G6P	*r_0534*	1.00	1.00	1.00
	x_2	G6P → F6P	*r_0467*	0.49	0.49	0.64
	x_3	G6P →→→ Ru5P + CO_2	*r_0466*	0.27	0.37	0.22
	x_4	F6P →→ G3P + DHAP	*r_0886 + r_0887*	0.63	0.63	0.69
		DHAP → G3P	*r_1054*	0.63	0.63	0.69
	x_5	Ro5P + X5P → S7P + G3P	*r_1049*	0.09	0.12	0.07
	x_6	E4P + X5P → F6P + G3P	*r_1050*	0.06	0.09	0.04
	x_7	S7P + G3P → F6P + E4P	*r_1048 + r_0887*	0.09	0.12	0.07
	x_8	3PG →→ PEP	*r_0893*	1.26	1.30	1.35
	x_9	PEP → PYR	*r_0962*	1.28	1.24	1.30
	x_{24}	PYR → ACA + CO_2	*r_0959*	0.07	0.02	0.20
	x_{18}	ACA → AC	*r_0173 + r_2116*	0.07	0.02	0.20
	x_{17}	AC + CoA → AcCoA	*r_0112*	0.07	0.02	0.21
	x_{16}	PYR + CO_2 → OAA	*r_0958 + r_2117 + r_2119_REV*	0.33	0.28	0.14
	x_{15}	OAA → PEP + CO_2	*r_0884*	0.08	0.00	0.00
Mitochondrial	x_{10}	PYR + CoA → AcCoA + CO_2	*r_0961*	0.74	0.82	0.89
	x_{11}	OAA + AcCoA → CIT + CoA	*r_0300*	0.71	0.75	0.81
	x_{12}	CIT →→→ AKG + CO_2	*r_0658 + r_2131*	0.71	0.75	0.63
	x_{13}	AKG →→→ SUCC + CO2	*r_0832*	0.60	0.64	0.52
		SUCC →→ MAL	*r_1021*	0.60	0.65	0.72
	x_{14}	MAL → PYR + CO_2	*r_0719 + r_0718*	0.03	0.02	0.07
Transport	x_{25}	glucose uptake (extra→cyt)	*r_1166*	1.00	1.00	1.00
	x_{23}	PYR transport (cyt→mit)	*r_2034 + r_1138_REV*	0.88	0.95	0.96
	x_{21}	OAA transport (cyt→mit)	*r_1239 + r_2132 + r_1126_REV + r_1226*	0.65	0.13	0.41
	x_{22}	OAA transport (mit→cyt)	*r_1126 + r_2132_REV*	0.50	0.00	0.00
Sink	x_{29}	G6P (cytoplasm)	*r_0195 + r_0758 + r_0888*	0.24	0.15	0.14
	x_{30}	Ro5P (cytoplasm)	*r_0916*	0.03	0.03	0.03
	x_{31}	E4P (cytoplasm)	*r_1708*	0.03	0.03	0.03
	x_{32}	3PG (cytoplasm)	*r_0891*	0.07	0.07	0.07
	x_{33}	PEP (cytoplasm)	*r_0065 - r_1127*	0.05	0.06	0.06
	x_{34}	OAA (cytoplasm)	*- r_0216*	0.10	0.14	0.09
	x_{35}	AcCoA (cytoplasm)	*r_0109 + r_0549 + r_0760 + r_2140 + r_2141*	0.07	0.02	0.02
	x_{36}	AcCoA (mitochondria)	*r_0025 + 2 • r_0104 + r_0560 + r_1838*	0.03	0.07	0.07
	x_{37}	PYR (mitochondria)	*r_0016 + 2 • r_0097*	0.18	0.14	0.14
	x_{38}	AKG (mitochondria)	*r_1838 - r_0118 - r_1099 + r_1088 - r_1112 - r_0217 + r_0664*	0.11	-0.02	0.11

<u>Abbreviations</u>: G6P: glucose 6-phosphate. F6P: fructose 6-phosphate. Ru5P: ribulose 5-phosphate. G3P: glyceraldehyde 3-phosphate. DHAP: dihydroxyacetone phosphate. X5P: xylulose 5-phosphate. Ro5P: ribose 5-phosphate. S7P: sedoheptulose 7-phosphate. E4P: erythrose 4-phosphate. 3PG: 3-phosphoglycerate. PEP: phosphoenolpyruvate. PYR: pyruvate. ACA: acetaldehyde. AC: acetate. AcCoA: acetyl-CoA. OAA: oxaloacetate. CIT: citrate. AKG: α-ketoglutarate. SUCC: succinate. MAL: malate.

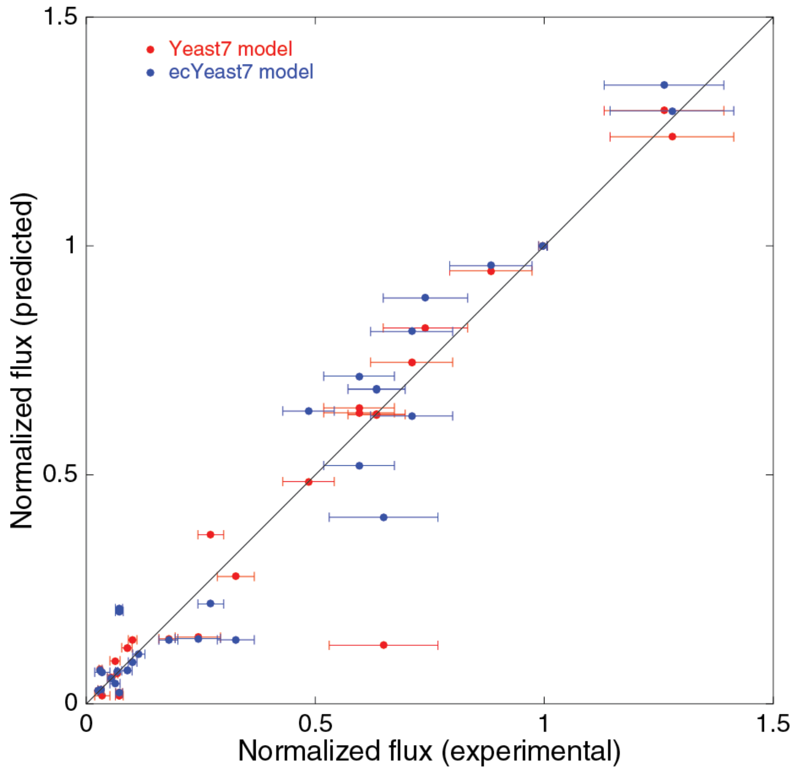

Figure S12: Performance of both Yeast7 (red) and ecYeast7 (blue) predicting intracellular flux ratios from central carbon metabolism. Experimental values obtained from a published ^{13}C-MFA study[50].

Finally, Figure S13A shows that increased flux variability by including enzyme constraints only affected a minority of fluxes, and the increase was no more than 2 mmol/gDWh. Figure S13B on the other hand shows all 3177 reduced fluxes, showing that most of them are reduced in over 90%.

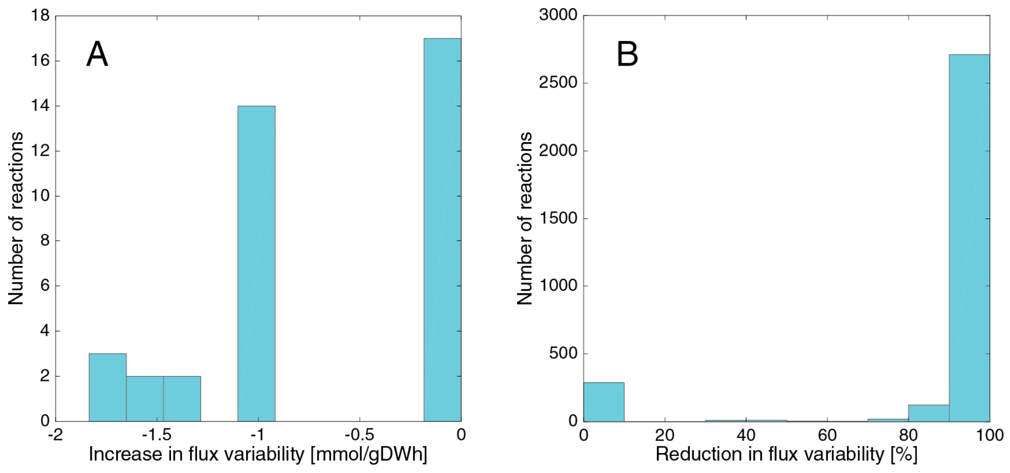

Figure S13: Histogram of reactions with (A) increased and (B) decreased flux variability.

4.7. *NDI1* knockout study

Figure S14 shows the exchange flux predictions of ecYeast7 for aerobic glucose limited chemostats of CEN.PK113-7D, for both the wild-type (Figure S14A) and the NDI1 knockout (Figure S14B). Compared to the experimental data[40], we see that the enzyme-constrained model adequately predicts a change in the critical growth rate.

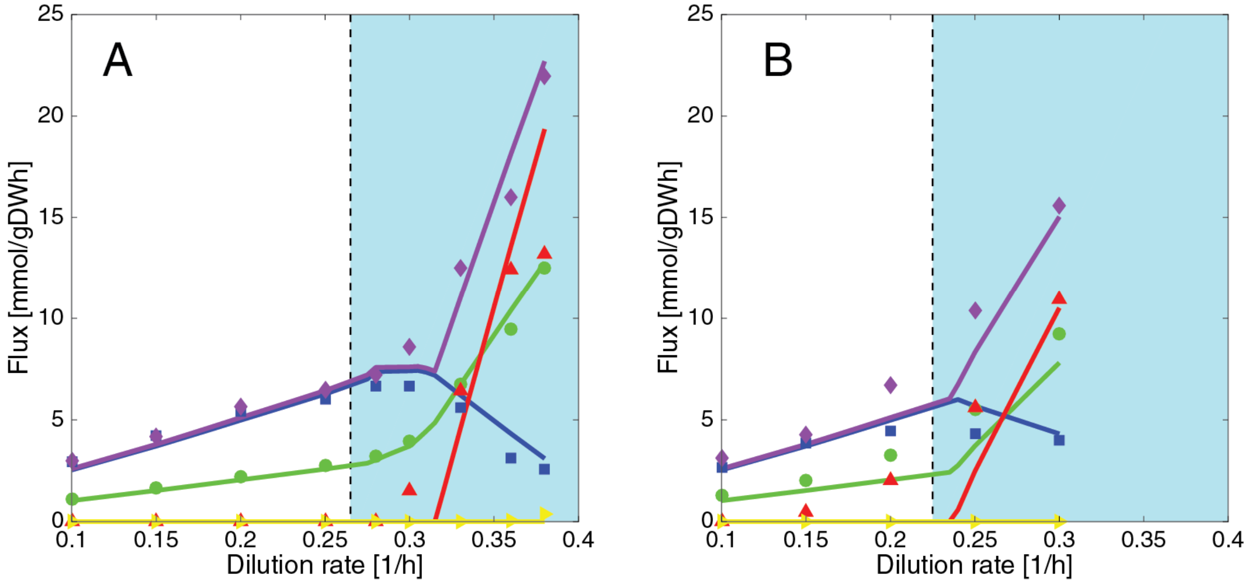

Figure S14: Model predictions (lines) and experimental values (points) for exchange fluxes from glucose-limited chemostats: glucose (green circles) and oxygen (blue squares) consumption, and CO_2 (purple diamonds), ethanol (red triangles) and glycerol (yellow right-pointing triangles) production. The light blue area denotes the region of both glucose and protein limitation. (A) Wild-type strain. (B) NDI1 knockout.

4.8. Flux control coefficients for farnesene production

As Figure S15 shows, the 2 most influential enzymes on the production of farnesene are HMG-CoA reductase (HMG1) and farnesene synthase (AFS1). Both of those enzymes are acting as major bottlenecks in the system, and therefore are interesting candidates to improve activity in order to increase farnesene production. The FCC of HMG-CoA reductase is approximately 3 times higher than the FCC from farnesene synthase given that they have approximately the same properties (molecular weight and k_{cat} value), but HMG-CoA reductase is needed 3 times for every farnesene molecule produced, whereas farnesene synthase is needed only once.

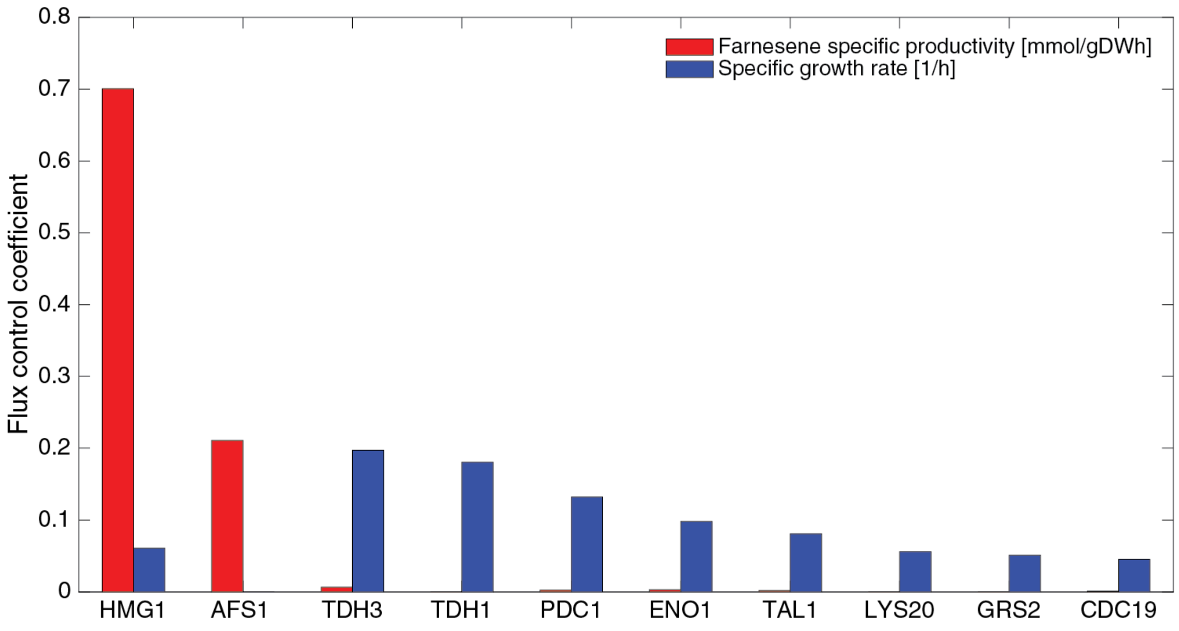

Figure S15: Flux control coefficients of the top 10 most influential enzymes in the model.

5. Supplementary references

1 J. D. Orth, I. Thiele and B. Ø. Palsson, *Nat. Biotechnol.*, 2010, **28**, 245–248.

2 C. Zhang, B. Ji, A. Mardinoglu, J. Nielsen and Q. Hua, *Bioinformatics*, 2015, **31**, 2324–2331.

3 H. W. Aung, S. A. Henry and L. P. Walker, *Ind. Biotechnol.*, 2013, **9**, 215–228.

4 J. Schellenberger, R. Que, R. M. T. Fleming, I. Thiele, J. D. Orth, A. M. Feist, D. C. Zielinski, A. Bordbar, N. E. Lewis, S. Rahmanian, J. Kang, D. R. Hyduke and B. Ø. Palsson, *Nat. Protoc.*, 2011, **6**, 1290–1307.

5 R. Agren, L. Liu, S. Shoaie, W. Vongsangnak, I. Nookaew and J. Nielsen, *PLoS Comput. Biol.*, 2013, **9**, e1002980.

6 I. Schomburg, A. Chang, S. Placzek, C. Söhngen, M. Rother, M. Lang, C. Munaretto, S. Ulas, M. Stelzer, A. Grote, M. Scheer and D. Schomburg, *Nucleic Acids Res.*, 2013, **41**, D764-72.

7 A. Nilsson and J. Nielsen, *Sci. Rep.*, 2016, **6**, 22264.

8 B. Boeckmann, *Nucleic Acids Res.*, 2003, **31**, 365–370.

9 M. Kanehisa and S. Goto, *Nucleic Acids Res.*, 2000, **28**, 27–30.

10 J. Förster, I. Famili, B. Ø. Palsson and J. Nielsen, *Genome Res.*, 2003, 244–253.

11 P. Magnelli, J. F. Cipollo and C. Abeijon, *Anal. Biochem.*, 2002, **301**, 136–50.

12 C. Verduyn, A. H. Stouthamer, W. A. Scheffers and J. P. van Dijken, *Antonie Van Leeuwenhoek*, 1991, **59**, 49–63.

13 S. J. Ferguson, *Proc. Natl. Acad. Sci.*, 2010, **107**, 16755–16756.

14 J. K. Stoops and S. J. Wakil, *Proc. Natl. Acad. Sci. U. S. A.*, 1980, **77**, 4544–4548.

15 H.-P. Huang and E. Cabib, *J. Biol. Chem.*, 1974, **249**, 3851–3857.

16 J. G. Hofler, C. J. Decedue, G. H. Luginbuhl, J. A. Reynolds and R. O. Burns, *J. Biol. Chem.*, 1975, **250**, 877–882.

17 F. J. Schendel, E. Mueller, J. Stubbe, A. Shiau and J. M. Smith, *Biochemistry*, 1989, **28**,

2459–2471.

18 I. F. Durr and H. Rudney, *J. Biol. Chem.*, 1960, **235**, 2572–8.

19 D. L. Bartlett, C. H. King and C. D. Poulter, *Methods Enzymol.*, 1985, **110**, 171–84.

20 D. K. Marvil and T. Leisinger, *J. Biol. Chem.*, 1977, **252**, 3295–303.

21 Y. Liu, R. Heeswijck, P. Hoj and N. Hoogenraad, *Eur. J. Biochem.*, 1995, **228**, 291–296.

22 N. Biswas, *Biochim. Biophys. Acta - Gen. Subj.*, 1996, **1290**, 95–100.

23 A. Breda, L. K. B. Martinelli, C. V Bizarro, L. A. Rosado, C. B. Borges, D. S. Santos and L. A. Basso, *PLoS One*, 2012, **7**, e39245.

24 A. Mitchell and B. Magasanik, *J. Biol. Chem.*, 1983, **258**, 119–124.

25 P. J. White, G. Millar and J. R. Coggins, *Biochem. J.*, 1988, **251**, 313–322.

26 M. Strassman and L. N. Ceci, *J. Biol. Chem.*, 1966, **241**, 5401–7.

27 M. R. Mejillano, H. Jahansouz, T. O. Matsunaga, G. L. Kenyon and R. H. Himes, *Biochemistry*, 1989, **28**, 5136–5145.

28 P. D. Pawelek, M. Allaire, M. Cygler and R. E. MacKenzie, *Biochim. Biophys. Acta - Protein Struct. Mol. Enzymol.*, 2000, **1479**, 59–68.

29 B. V Ramasastri and R. L. Blakley, *J. Biol. Chem.*, 1962, **237**, 1982–8.

30 H. Hirsch and D. M. Greenberg, *J. Biol. Chem.*, 1967, **242**, 2283–2287.

31 F. Ramos, M. Guezzar, M. Grenson and J.-M. Wiame, *Eur. J. Biochem.*, 1985, **149**, 401–404.

32 S. B. Inoue, N. Takewakt, T. Takasuka, T. Mio, M. Adachi, Y. Fujii, C. Miyamoto, M. Arisawa, Y. Furuichi and T. Watanabe, *Eur. J. Biochem.*, 1995, **231**, 845–854.

33 M. Wang, M. Weiss, M. Simonovic, G. Haertinger, S. P. Schrimpf, M. O. Hengartner and C. von Mering, *Mol. Cell. Proteomics*, 2012, **11**, 492–500.

34 T. L. Nissen, U. Schulze, J. Nielsen and J. Villadsen, *Microbiology*, 1997, **143**, 203–218.

35 R. Adadi, B. Volkmer, R. Milo, M. Heinemann and T. Shlomi, *PLoS Comput. Biol.*, 2012, **8**.

36 Q. K. Beg, A. Vazquez, J. Ernst, M. A. de Menezes, Z. Bar-Joseph, A. L. Barabási and Z. N. Oltvai, *Proc. Natl. Acad. Sci. U. S. A.*, 2007, **104**, 12663–12668.

37 A. Bar-Even, E. Noor, Y. Savir, W. Liebermeister, D. Davidi, D. S. Tawfik and R. Milo, *Biochemistry*, 2011, **50**, 4402–4410.

38 E. W. Dijkstra, *Numer. Math.*, 1959, **1**, 269–271.

39 B. J. Sánchez and J. Nielsen, *Integr. Biol.*, 2015, **7**, 846–858.

40 M. A. H. Luttik, B. M. Bakker, C. Bro and P. Ko, 2000, **182**, 4730–4737.

41 P. Van Hoek, J. P. Van Dijken and J. T. Pronk, *Appl. Environ. Microbiol.*, 1998, **64**, 4226–4233.

42 N. E. Lewis, K. K. Hixson, T. M. Conrad, J. A. Lerman, P. Charusanti, A. D. Polpitiya, J. N. Adkins, G. Schramm, S. O. Purvine, D. Lopez-Ferrer, K. K. Weitz, R. Eils, R. König, R. D. Smith and B. Ø. Palsson, *Mol. Syst. Biol.*, 2010, **6**, 390.

43 P.-J. Lahtvee, R. Kumar, B. Hallström and J. Nielsen, *Mol. Biol. Cell*, 2016, **27**, 2505–2514.

44 R. Pereira, J. Nielsen and I. Rocha, *Metab. Eng. Commun.*, 2016, **3**, 153–163.

45 J. P. Van Dijken, J. Bauer, L. Brambilla, P. Duboc, J. M. Francois, C. Gancedo, M. L. F. Giuseppin, J. J. Heijnen, M. Hoare, H. C. Lange, E. A. Madden, P. Niederberger, J. Nielsen, J. L. Parrou, T. Petit, D. Porro, M. Reuss, N. Van Riel, M. Rizzi, H. Y. Steensma, C. T. Verrips, J. Vindel??v and J. T. Pronk, *Enzyme Microb. Technol.*, 2000, **26**, 706–714.

46 E. Boles and C. P. Hollenberg, *FEMS Microbiol. Rev.*, 1997, 21, 85–111.

47 R. Mahadevan and C. H. Schilling, *Metab. Eng.*, 2003, **5**, 264–276.

48 S. Bordel, R. Agren and J. Nielsen, *PLoS Comput. Biol.*, 2010, **6**, 16.

49 P.-J. Lahtvee, B. J. Sánchez, A. Smialowska, S. Kasvandik, I. E. Elsemman, F. Gatto and J. Nielsen, *Cell Syst.*, 2017, **4**, 1–10.

50 P. Jouhten, E. Rintala, A. Huuskonen, A. Tamminen, M. Toivari, M. Wiebe, L. Ruohonen, M. Penttilä and H. Maaheimo, *BMC Syst. Biol.*, 2008, **2**.
